# First Total Synthesis of Varioxiranol A

**DOI:** 10.3390/molecules24050862

**Published:** 2019-02-28

**Authors:** Angelika Lásiková, Jana Doháňošová, Mária Štiblariková, Martin Parák, Ján Moncol, Tibor Gracza

**Affiliations:** 1 Department of Organic Chemistry, Slovak University of Technology in Bratislava, Radlinského 9, 812 37 Bratislava, Slovakia; maria.stiblarikova@stuba.sk (M.Š.); xparakm@is.stuba.sk (M.P.); tibor.gracza@stuba.sk (T.G.); 2 Central Laboratories, Slovak University of Technology in Bratislava, Radlinského 9, 812 37 Bratislava, Slovakia; jana.dohanosova@stuba.sk; 3 Department of Inorganic Chemistry, Slovak University of Technology in Bratislava, Radlinského 9, 812 37 Bratislava, Slovakia; jan.moncol@stuba.sk

**Keywords:** synthesis of natural products, varioxiranol A, 4-*epi*-varioxiranol A, absolute structure, *Emericella variecolor*

## Abstract

The paper describes the first total synthesis of natural varioxiranol A by chiral pool approach and confirmation of its absolute configuration by single-crystal X-ray analysis. The target varioxiranol A and its 4-epimer were obtained after 10 steps from single and available chiral source 1,2-*O*-isopropylidene-d-glyceraldehyde in an overall yield of 10% and 6%, respectively. A synthetic strategy based on the Julia–Kocieński coupling reaction between aromatic sulfone and corresponding aldose derivative makes it possible to prepare other interesting polyketide derivatives (varioxiranols B-G, varioxirane, varioxiranediols).

## 1. Introduction

**Figure 1:**
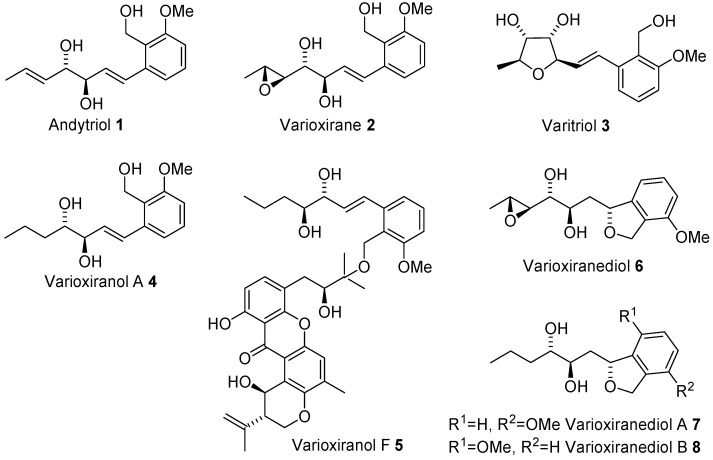
Natural compounds isolated from a strain of *Emericella variecolor* **1**–**8**.

The fungus *Emericella variecolor* [1] is considered as a promising source of interesting bioactive compounds. During the past several decades, the large number of natural products of this origin had been isolated and evaluated for their diversiform biological activities. First, Dunn and Johnstone [2] isolated from a static culture of a pure strain of the fungus *Aspergillus variecolor* (imperfect state of *Emericella variecolor*) 2-methoxy-6-(3,4-dihydroxyhepta-1,5-dienyl) benzyl alcohol **1** (Figure 1) along with other metabolites-6-methoxymellein, siderin, andibenin, and andilesin A–C. The structure of **1** was established by NMR spectroscopy [3], absolute configuration (3*R*,4*S*) was later determined by total synthesis [4], and the trivial name “andytriol” was kindly suggested by prof. Johnstone and used for the first time in the manuscript dealing with the synthesis [4]. In 2002, Malmstrøm et al. [5] reported the isolation of benzyl alcohols (varitriol **3**, varioxirane **2**), prenylxanthones (shamixanthone, varixanthone, tajixanthone), and cyclopentanones from a strain of *E. variecolor* derived from a Caribbean sponge. In particular, **3** showed notably increased potency toward selected renal, CNS, and breast cancer cell lines. The authors also proposed a hypothetical biogenetic relationship between these products via enzymatic intramolecular S_N_2 epoxide ring opening and pointed out that natural andytriol **1** could be involved in this biosynthetic pathway to **3** via epoxide **2**. Recently, seven new polyketide derivatives with benzyl alcohol structural motif, namely varioxiranols A–G, isolated by chemical examination of a sponge (*Cinachyrella* sp.)-associated *E. variecolor* fungus and tested for lipid-lowering effects against oleic acid, elicited lipid accumulation in HepG2 liver cells. Among these secondary metabolites, varioxiranol A **4** exerted inhibition activity and showed no toxicity [6].

Interesting activity against drug-resistant microbial pathogens was observed by varioxiranediols **6** and **7**. These metabolites of formal cyclisation of varioxirane **2** and varioxiranol A **4** were isolated from the same endophytic fungus. The structure and absolute configuration of **6** [7] and varioxiranediols A **7** and B **8** [8] were confirmed by the X-ray analysis supporting the structural relationship of the isolated natural compounds. 

In the course of our long-term program directed towards the synthesis of natural compounds and secondary metabolites isolated from *E. variecolor*, we have developed the synthesis of varitriol **3** [9,10,11,12,13], andytriol **1** [4], and varioxirane **2** [4] and examined their antitumor activity. Herein, we describe the first total synthesis of natural varioxiranol A **4** and its 4-epimer **9** that should also be general route for the synthesis of all other varioxiranols, varioxirane, and varioxiranediols.

## 2. Results and Discussion

**Scheme 1:**
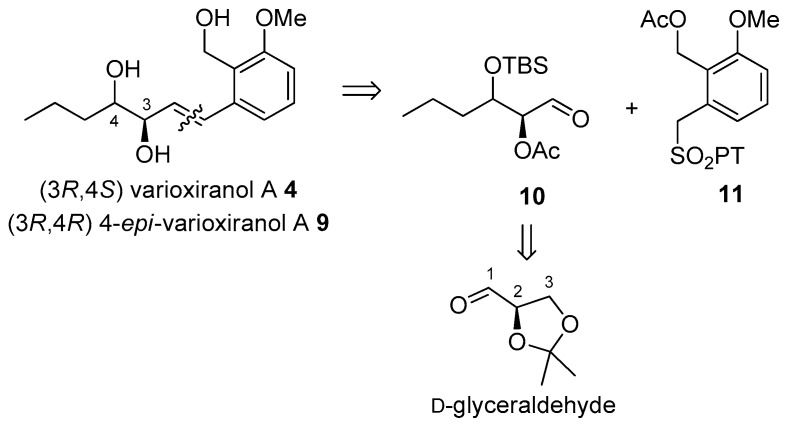
Retrosynthetic analysis of **4** and **9**.

The synthetic strategy takes advantage of our previous synthesis of andytriol **1**. Target compounds could be readily obtained by coupling of the known sulfone **11** [4] bearing benzyl alcohol moiety with corresponding aldehydes via Julia–Kocieński olefination (Scheme 1). The aldehydic partner for the olefination, dihydroxyhexanal **10**, having the configuration of natural enantiomer **4** and its 4-epimer **9** at C-2, could be accessible from d-glyceraldehyde by introduction of the propyl group at C-1 followed by the oxidation of carbon at the other end (C-3).

**Scheme 2:**
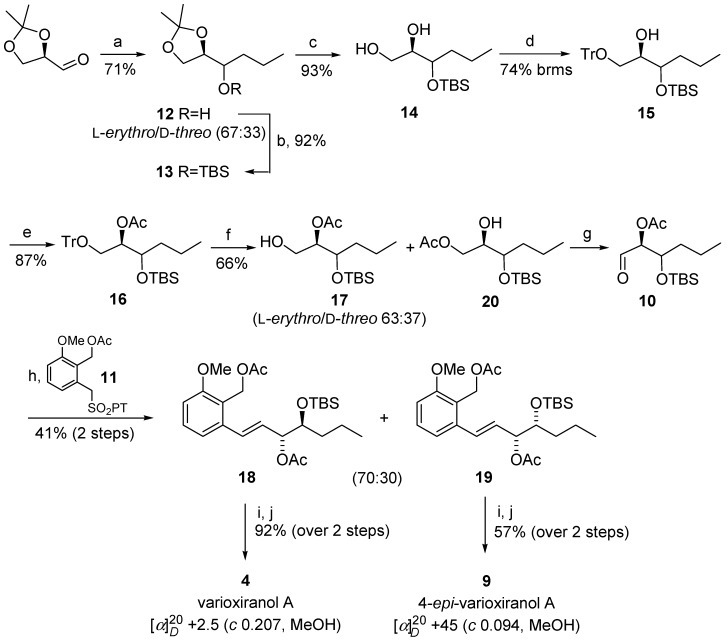
Synthesis of natural varioxiranol A **4** and 4-*epi*-varioxiranol A **9**. Reagents and conditions: (**a**) prop-1-ylmagnesium chloride, THF, Et_2_O, r.t., 1 h; (**b**) TBSCl, imidazole, CH_2_Cl_2_, 0 °C to r.t., 23 h; (c) TFA (50%), CH_2_Cl_2_, r.t., 1 h; (**d**) TrCl, Et_3_N, DMAP, CH_2_Cl_2_, 0 °C to r.t., 15 h; (**e**) Ac_2_O, DMAP, CH_2_Cl_2_, r.t., 30 min; (**f**) HCOOH/Et_2_O (1/1), r.t., 50 min; (**g**) DMSO, (COCl)_2_, Et_3_N, CH_2_Cl_2_, −78 °C to r.t., 2.5 h; (**h**) KHMDS, dimethoxyethane, sulfone **11**, CH_2_Cl_2_, −60 °C to r.t., 40 min; (**i**) K_2_CO_3_, MeOH, r.t. 2.5 h; (**j**) TBAF × 3H_2_O, THF, 0 °C to r.t., 4.5 h.

The synthesis of varioxiranols A **4** commenced from isopropylidene-d-glyceraldehyde, a commercially available starting material, or readily obtainable from d-mannitol via the route using standard carbohydrate chemistry [14]. The required six-carbon chain of the key fragment **10** [15] was obtained by the Grignard addition of propylmagnesium chloride to isopropylidene-d-glyceraldehyde in THF/Et_2_O [15] (Scheme 2). A diastereomeric mixture of partially protected l-*erythro/*d-*threo* hexenetriols **12** was isolated in the ratio of 67:33 with 71% yield. To prepare both epimers of varioxiranol A **4**, we continued synthesis with the mixture of diastereomers **12**. The aldehydic partner for the Julia–Kocieński coupling, hexenose derivative **10** was prepared using a selective protection–deprotection sequence. Firstly, free hydroxyl group of **12** was protected as *tert*-butyldimethylsilylether **13** with good yield (92%). Acidic hydrolysis of **13** with trifluoroacetic acid in dichloromethane afforded vicinal diol **14** (93%) which was selectively tritylated on terminal hydroxyl group to give **15**. Subsequent acetylation of **15** provided fully protected triol **16** (87%). Selective deprotection of primary hydroxy group was then achieved by smooth tritylether hydrolysis using formic acid in ether furnishing alcohol **17 [16]**, however, as an inseparable mixture along with the product of the acetyl group migration **20** taking place even during MPLC. Swern oxidation of primary hydroxyl group under conventional reaction conditions provided desired aldehyde **10**, which was used in the next step without further purification. The crude aldehyde was subjected to Julia–Kocieński coupling with 2-methoxy-6-[(1-phenyl-1*H*-tetrazol-5-ylsulfonyl)methyl]benzyl acetate **11**, which was prepared according to the literature [4]. Thus, potassium hexamethyldisilazane was added to a nearly equimolar mixture of sulfone **11** and aldehyde **10** in dimethoxyethane at −60 °C and stirred for 40 min at room temperature affording coupling products **18**/**19** in 41% yield (in ratio 70:30) and with excellent *E*-selectivity. The resulting alkenes **18**/**19** could be separated by MPLC and preparative TLC. The final steps, removal of all protecting groups, were run in parallel with the pure diastereomers **18** and **19**. The first, basic hydrolysis of acetyl groups with K_2_CO_3_ in MeOH, was followed by treatment of the crude mixture with TBAF in THF and finally, flash chromatography purification furnished the target compounds (+)-**4** and **9** in 92% and 57% yield, respectively, over the last two steps. The ^1^H and ^13^C NMR, HRMS spectra, and the specific rotation {[α]${}_{D}^{20}$ +2.5 (*c* 0.207, MeOH), lit. [2], [α]${}_{D}^{20}$ +5.8 (*c* 0.53, MeOH)} of synthetic varioxiranol A **4** were in good agreement with the reported data for the natural product.

**Figure 2:**
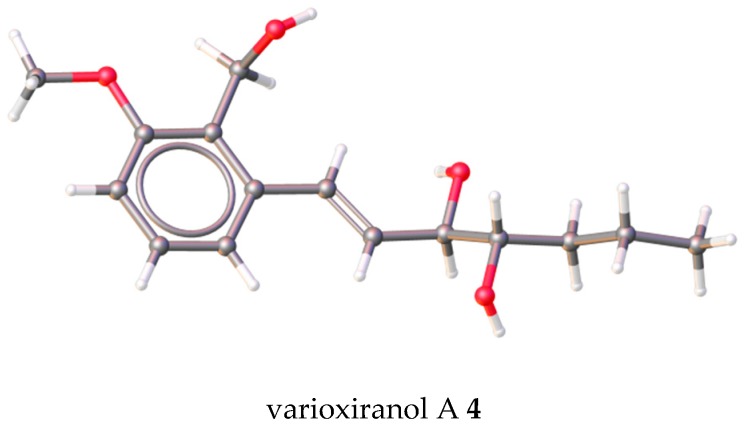

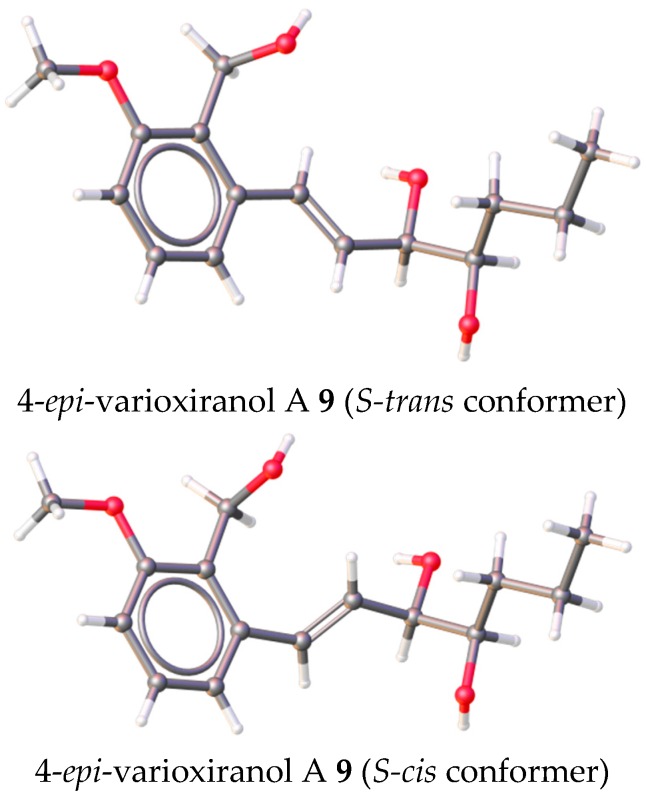
A ball-and-stick view of crystal structures **4** and **9**.

Definitive confirmation of absolute configuration of the target compound has been provided by single-crystal X-ray analysis. An X-ray study of both epimers confirmed 3*R*,4*S* (*anti*) configuration of the natural varioxiranol A **4** and 3*R*,4*R* (*syn*) configuration of its 4-epimer **9** (Figure 2). Interestingly, the crystal lattice of the 4-epimer **9** is composed of *S-cis* and *S-trans* conformers (two crystallographic independent molecules in cell; see Supplementary Materials).

## 3. Experimental Section

### 3.1. General Methods

Commercial reagents were used without further purification. All solvents were distilled before use. Hexanes refer to the fraction boiling at 60–65 °C. Flash column liquid chromatography (FLC) was performed on silica gel Kieselgel 60 (40–63 μm, 230–400 mesh, Merck, Darmstadt, Germany) and analytical thin-layer chromatography (TLC) was performed on aluminium plates precoated with either 0.2 mm (DC-Alufolien, Merck) or 0.25 mm silica gel 60 F_254_ (ALUGRAM^®^ SIL G/UV_254_, Macherey-Nagel, Fisher Scientific, Loughborough, UK). The compounds were visualized by UV fluorescence and by dipping the plates in an aqueous H_2_SO_4_ solution of cerium sulphate/ammonium molybdate followed by charring with a heat gun. Melting points were obtained using a Boecius apparatus (Büchi® melting point apparatus Model B-545, BÜCHI Labortechnik AG, Flawil, Switzerland) and are uncorrected. Optical rotations were measured with a JASCO P-2000 polarimeter (JASCO, Easton, MD, USA) and are given in units of 10^−1^ deg.cm^2^.g^−1^. FTIR spectra were obtained on a Nicolet 5700 spectrometer (Thermo Electron, Thermo Fisher Scientific, Waltham, MA, USA) equipped with a Smart Orbit (diamond crystal ATR) accessory, using the reflectance technique (4000–400 cm^−1^).^1^H and ^13^C NMR spectra were recorded on either 300 (75) MHz or 600 (150) MHz Varian spectrometer (Varian Inc., Palo Alto, CA, USA). Chemical shifts (*δ*) are quoted in ppm and are referenced to tetramethylsilane (TMS, *δ* = 0 ppm) as internal standard for ^1^H NMR and to CDCl_3_ peak (*δ* = 77.16 ppm in case of ^13^C NMR). High-resolution mass spectra (HRMS) were recorded on an OrbitrapVelos mass spectrometer (Thermo Scientific, Waltham, MA, USA; Bremen, Germany) with a heated electrospray ionisation (HESI) source. The mass spectrometer was operated with full scan (50–2000 amu) in positive or negative FT mode (at a resolution of 100,000). The analyte was dissolved in methanol and infused via syringe pump at a rate of 5 mL/min. The heated capillary was maintained at 275 °C with a source heater temperature of 50 °C and the sheath, auxiliary, and sweep gases were at 10, 5, and 0 units, respectively. Source voltage was set to 3.5 kV.

Data collection and cell refinement of **4** and **9** were made on a Stoe StadiVari diffractometer (Stoe & Cie GmbH, Darmstadt, Germany) using a Pilatus3R 300K HPAD detector and the microfocus source Xenocs Genix3D Cu HF (λ = 1.54186 Å). The structures were solved using SHELXT [17] and refined by the full-matrix least-squares procedure with SHELXL (ver. 2018/3) (for **4**) [18] or CRYSTALS (ver. 14.61) (**9**) [19]. The structures were drawn using the OLEX2 package [20]. The absolute configurations of both compounds were determined. The Flack parameter x = −0.08(5) for **9** was calculated by Parsons method [21]. The absolute structure of very small crystal of **4** is impossible to determine based on the Flack parameter, however, using Hooft parameter [22] with (Gaussian) statistics led to the conclusive value of y = 0.07(11). The deposition numbers CCDC 1892452 (**4**) and CCDC 1892453 (**9**) contain the supplementary crystallographic data for this paper. These data can be obtained free of charge from http://www.ccdc.cam.ac.uk/conts/retrieving.html (or from the CCDC, 12 Union Road, Cambridge CB2 1EZ, UK.

### 3.2. (2R,3S)-1,2-O-Isopropylidene-hexane-1,2,3-triol (l-erythro-12) and (2R,3R)-1,2-O-isopropylidene-hexane-1,2,3-triol (d-threo-12)

A solution of propylmagnesium chloride in diethyl ether (2.0 M solution, 5.8 mL, 11.6 mmol) was added dropwise to a stirred solution of freshly distilled 1,2-*O*-isopropylidene-d-glyceraldehyde (1 g, 7.68 mmol) in dry THF (62 mL) at room temperature. Following the addition, the reaction mixture was stirred for 1 h (TLC control). The reaction was quenched by pouring into a sat. aqueous NH_4_Cl (62 mL), the aqueous layer was extracted with diethyl ether (3 × 35 mL), and the combined organic layers were dried and concentrated. The residue was purified by MPLC (gradient AcOEt/hexanes 0/100 to 30/70) to give **12** (954 mg, 71%, l-erythro-**12**/d-threo-**12** 67:33) as colorless liquid. ^1^H NMR (600 MHz, CDCl_3_) δ (l-erythro-**12**) 0.95 (t, H-6, *J* = 7.1 Hz, 3H), 1.33–1.44 (m, H-4, H-5a, 2 × CH_3_, 9H), 1.51–1.59 (m, H-5b, 1H), 1.96 (d, OH, *J* = 2.9 Hz, 1H), 3.78–3.82 (m, H-3, 1H), 3.91 (dd, H-1a, *J* = 7.3, 8.0 Hz, 1H), 3.97 (dd, H-1b, *J* = 6.5, 8.0 Hz, 1H), 4.04 (ddd, H-2, *J* = 4.0, 6.5, 7.2 Hz, 1H); δ (D-threo-**12**) 0.94 (t, H-6, *J* = 7.2 Hz, 3H), 1.30–1.36 (m, H-4a, 1H), 1.37 (s, CH_3_, 3H), 1.37–1.44 (m, H-5a, 1H), 1.44 (s, CH_3_, 3H), 1.44–1.48 (m, H-4b, 1H), 1.53–1.59 (m, H-5b, 1H), 2.15 (d, OH, *J* = 5.2 Hz, 1H), 3.48-3.53 (m, H-3, 1H), 3.73 (dd, H-1a, *J* = 6.3, 7.7 Hz, 1H), 3.96–4.00 (m, H-2, 1H), 4.02 (dd, H-1b, *J* = 6.6, 7.8 Hz, 1H); ^13^C NMR (150 MHz, CDCl_3_) δ (l-erythro-**12**) 14.2 (C-6), 19.1 (C-5), 25.5 (CH_3_), 26.6 (CH_3_), 34.8 (C-4), 64.6 (C-1), 70.5 (C-3), 78.8 (C-2), 109.0 (C_q_); δ (D-threo-**12**) 14.2 (C-6), 18.9 (C-5), 25.5 (CH_3_), 26.8 (CH_3_), 36.0 (C-4), 66.3 (C-1), 72.2 (C-3), 79.3 (C-2), 109.5 (C_q_); HRMS (ESI) calcd for C_9_H_18_O_3_Na^+^ [M + Na]^+^: 197.1148, found: 197.1148.

### 3.3. (2R,3S)-1,2-O-Isopropylidene-3-O-tert-butyldimethylsilyl-hexane-1,2,3-triol (l-erythro-13) and (2R,3R)-1,2-O-isopropylidene-3-O-tert-butyldimethylsilyl-hexane-1,2,3-triol (d-threo-13)

Imidazole (1.09 g, 16.0 mmol) was added to a solution of diastereomeric mixture **12** (928 mg, 5.33 mmol) in dry CH_2_Cl_2_ (11 mL) at room temperature. The mixture was subsequently cooled to 0 °C and tert-butyldimethylsilyl chloride (1.61 g, 10.7 mmol) was added. The reaction mixture was then stirred for 23 h at room temperature. After the dilution with CH_2_Cl_2_ (140 mL), the reaction mixture was washed with water (2 × 140 mL), the water phase was extracted with CH_2_Cl_2_ (3 × 90 mL), and combined organic layers were dried and concentrated. The residue was purified by MPLC (isocratic AcOEt/hexanes 2/98) to afford **13** (1.42 g, 92%, l-erythro-**13**/d-threo-**13** 67:33) as colorless liquid. ^1^H NMR (600 MHz, CDCl_3_) δ (l-erythro-**13**) 0.06 (s, CH_3_, 3H), 0.07 (s, CH_3_, 3H), 0.88 (s, tBu, 9H), 0.91 (t, H-6, *J* = 7.3 Hz, 3H), 1.34 (s, CH_3_, 3H), 1.35–1.47 (m, H-4a, H-5, CH_3_, 6H), 1.49–1.52 (m, H-4b, 1H), 3.73–3.76 (m, H-3, 1H), 3.80–3.84 (m, H-1a, 1H), 3.96–4.00 (m, H-1b, H-2c, 2H); δ (D-threo-**13**) 0.07 (s, CH_3_, 3H), 0.08 (s, CH_3_, 3H), 0.89 (s, tBu, 9H), 0.91 (t, H-6, *J* = 7.2 Hz, 3H), 1.30–1.39 (H-4, H-5a, CH_3_, 6H), 1.41 (s, CH_3_, 3H), 1.45-1.50 (m, H-5b, 1H), 3.69 (ddd, H-3, *J* = 4.2, 6.0, 7.7 Hz, 1H), 3.71 (dd, H-1a, *J* = 7.4, 8.2 Hz, 1H), 3.94 (dd, H-1b, *J* = 6.6, 8.2 Hz, 1H), 4.04 (ddd, H-2, *J* = 6.0, 6.6, 7.3 Hz, 1H); ^13^C NMR (150 MHz, CDCl_3_) δ (l-erythro-**13**) −4.2 (CH_3_), −4.1 (CH_3_), 14.5 (C-6), 17.8 (C-5), 18.2 (tBu), 25.6 (CH_3_), 26.0 (tBu), 26.8 (CH_3_), 37.0 (C-4), 66.6 (C-1), 72.4 (C-3), 78.4 (C-2), 109.0 (C_q_); δ (D-threo-**13**) −4.5 (CH_3_), −4.1 (CH_3_), 14.4 (C-6), 18.4 (tBu), 19.0 (C-5), 25.4 (CH_3_), 26.1 (tBu), 26.6 (CH_3_), 34.7 (C-4), 65.7 (C-1), 73.2 (C-3), 78.9 (C-2), 109.2 (C_q_); HRMS (ESI) calcd for C_15_H_32_O_3_SiNa^+^ [M + Na]^+^: 311.2013, found: 311.2013.

### 3.4. (2R,3S)-3-O-tert-Butyldimethylsilyl-hexane-1,2,3-triol (l-erythro-14) and (2R,3R)-3-O-tert-butyldimethylsilyl-hexane-1,2,3-triol (d-threo-14)

Trifluoroacetic acid (50%, 3.3 mL) was added dropwise to a vigorously stirred solution of compound **13** (1.39 g, 4.81 mmol) in CH_2_Cl_2_ (120 mL) at room temperature. The reaction mixture was stirred for 55 min, then diluted with CH_2_Cl_2_ (220 mL), and washed with sat. aqueous NaHCO_3_ (60 mL) and water (2 × 130 mL). The organic phase was dried and concentrated to give crude **14** (1.11 g, 93%, l-erythro-**14**/d-threo-**14** 67:33) as colorless oil, which was used in the next step without further purification. ^1^H NMR (600 MHz, CDCl_3_) δ (l-erythro-**14**) 0.09 (s, CH_3_, 3H), 0.11 (s, CH_3_, 3H), 0.89 (s, tBu, 9H), 0.91 (t, H-6, *J* = 7.3 Hz, 3H), 1.26–1.44 (m, H-4a, H-5, 3H), 1.52–1.58 (m, H-4b, 1H), 2.20 (bs, 2x OH, 2H), 3.60 (ddd, H-2, *J* = 3.5, 3.7, 5.5 Hz, 1H), 3.66 (dd, H-1a, *J* = 3.5, 11.5 Hz, 1H), 3.79 (dd, H-1b, *J* = 5.5, 11.5 Hz, 1H), 3.83 (ddd, H-3, *J* = 3.7, 5.6, 6.6 Hz, 1H); δ (D-threo-**14**) 0.08 (s, CH_3_, 3H), 0.09 (s, CH_3_, 3H), 0.90 (s, tBu, 9H), 0.92 (t, H-6, *J* = 7.3 Hz, 3H), 1.28–1.44 (m, H-4a, H-5, 3H), 1.61–1.68 (m, H-4b, 1H), 2.20 (bs, 2 × OH, 2H), 3.56-3.60 (m, H-1, H-2, 3H), 3.68 (ddd, H-3, *J* = 2.8, 4.6, 6.9 Hz, 1H); ^13^C NMR (150 MHz, CDCl_3_) δ (l-erythro-**14**) −4.5 (CH_3_), −4.4 (CH_3_), 14.4 (C-6), 18.2 (tBu), 18.7 (C-5), 26.0 (tBu), 35.8 (C-4), 63.4 (C-1), 73.2 (C-2), 75.3 (C-3); δ (D-threo-**14**) −4.6 (CH_3_), −4.0 (CH_3_), 14.4 (C-6), 18.2 (tBu), 18.4 (C-5), 26.0 (tBu), 36.2 (C-4), 64.7 (C-1), 72.4 (C-3), 73.1 (C-2); HRMS (ESI) calcd for C_12_H_28_O_3_SiNa^+^ [M + Na]^+^: 271.1700, found: 271.1700.

### 3.5. (2R,3S)-3-O-tert-Butyldimethylsilyl-1-O-trityl-hexane-1,2,3-triol (l-erythro-15) and (2R,3R)-3-O-tert-butyldimethylsilyl-1-O-trityl-hexane-1,2,3-triol (d-threo-15)

A solution of trityl chloride (1.01 g, 3.64 mmol) in dry CH_2_Cl_2_ (2.4 mL) was cooled to 0 °C, and triethylamine (0.95 mL, 6.84 mmol) and DMAP (46 mg, 0.37 mmol) were added. Subsequently, a solution of compound **14** (772 mg, 3.11 mmol) in CH_2_Cl_2_ (2.4 mL) was added dropwise. After warming to room temperature, the reaction mixture was stirred for 15 h, then quenched with sat. aqueous NH_4_Cl (25 mL), the aqueous layer was extracted with CH_2_Cl_2_ (3 × 10 mL), and the combined organic layers were dried and concentrated. The residue was purified by MPLC (gradient AcOEt/hexanes 0/100 to 5/95) to afford **15** (1.01 g, 74% brsm, l-erythro-**15**/d-threo-**15** 64:36) as colorless oil. ^1^H NMR (600 MHz, CDCl_3_) δ (l-erythro-**15**) −0.07 (s, CH_3_, 3H), 0.01 (s, CH_3_, 3H), 0.79 (s, tBu, 9H), 0.85 (t, H-6, *J* = 7.2 Hz, 3H), 1.21–1.28 (m, H-4a, H-5a, 2H), 1.36–1.47 (m, H-4b, H-5b, 2H), 2.36 (d, OH, *J* = 2.8 Hz, 1H), 3.17 (dd, H-1a, *J* = 7.7, 9.4 Hz, 1H), 3.23 (dd, H-1b, *J* = 4.5, 9.4 Hz, 1H), 3.69 (ddd, H-3, *J* = 4.1, 4.5, 6.9 Hz, 1H), 3.79-3.83 (m, H-2, 1H), 7.22–7.25 (m, Tr-H_p_, 3H), 7.28–7.32 (m, Tr-H_m_, 6H), 7.42–7.45 (m, Tr-H_o_, 6H); δ (D-threo-**15**) -0.13 (s, CH_3_, 3H), 0.02 (s, CH_3_, 3H), 0.78 (s, tBu, 9H), 0.90 (t, H-6, *J* = 7.2 Hz, 3H), 1.30-1.35 (m, H-4a, H-5, 3H), 1.58–1.63 (m, H-4b, 1H), 2.37 (d, OH, *J* = 7.5 Hz, 1H), 3.03 (dd, H-1a, *J* = 6.1, 9.4 Hz, 1H), 3.22 (dd, H-1b, *J* = 6.3, 9.4 Hz, 1H), 3.65–3.69 (m, H-2, 1H), 3.79-3.82 (m, H-3, 1H), 7.22–7.25 (m, Tr-H_p_, 3H), 7.27–7.31 (m, Tr-H_m_, 6H), 7.42–7.45 (m, Tr-H_o_, 6H); ^13^C NMR (150 MHz, CDCl_3_) δ (l-erythro-**15**) −4.4 (CH_3_), −4.3 (CH_3_), 14.4 (C-6), 18.2 (tBu), 18.3 (C-5), 26.0 (tBu), 34.5 (C-4), 65.1 (C-1), 73.3 (C-3), 73.5 (C-2), 86.9 (Trt-C_q_), 127.2 (3 × Tr-C_p_), 128.0 (6 × Tr-C_m_), 128.8 (6 × Tr-C_o_), 144.1 (3 × Tr-C_ipso_); δ (D-threo-**15**) −4.7 (CH_3_), −4.1 (CH_3_), 14.4 (C-6), 18.2 (tBu), 18.7 (C-5), 26.0 (tBu), 36.1 (C-4), 65.3 (C-1), 71.8 (C-2), 71.9 (C-3), 86.8 (Tr-C_q_), 127.1 (3 × Tr-C_p_), 127.9 (6 × Tr-C_m_), 128.8 (6 × Tr-C_o_), 144.2 (3 × Tr-C_ipso_); HRMS (ESI) calcd for C_31_H_42_O_3_SiNa^+^ [M + Na]^+^: 513.2795, found: 513.2795.

### 3.6. (2R,3S)-2-O-Acetyl-3-O-tert-butyldimethylsilyl-1-O-trityl-hexane-1,2,3-triol (l-erythro-16) and (2R,3R)-2-O-acetyl-3-O-tert-butyldimethylsilyl-1-O-trityl-hexane-1,2,3-triol (d-threo-16)

DMAP (771 mg, 6.32 mmol) and Ac_2_O (0.60 mL, 6.32 mmol) were added to a soluion of triol **15** (1.03 g, 2.11 mmol) in dry CH_2_Cl_2_ (30 mL) at room temperature. The reaction mixture was stirred for 30 min and quenched with sat. aqueous NaHCO_3_ (30 mL). The water phase was extracted with CH_2_Cl_2_ (3 × 40 mL), combined organic layers were dried and concentrated. The residue was purified by MPLC (gradient AcOEt/hexanes 0/100 to 5/95) to give **16** (975 mg, 87%, l-erythro-**16**/d-threo-**16** 65:35) as colorless oil. ^1^H NMR (600 MHz, CDCl_3_) δ (l-erythro-**16**) -0.10 (s, CH_3_, 3H), -0.04 (s, CH_3_, 3H), 0.74 (s, tBu, 9H), 0.85 (t, H-6, *J* = 7.1 Hz, 3H), 1.22–1.39 (m, H-4, H-5, 4H), 2.10 (s, C(O)CH_3_, 3H), 3.25–3.29 (m, H-1, 2H), 3.78 (ddd, H-3, *J* = 3.5, 5.0, 6.7 Hz, 1H), 5.15 (ddd, H-2, *J* = 3.5, 4.7, 6.8 Hz, 1H), 7.20–7.24 (m, Tr-H_p_, 3H), 7.27–7.30 (m, Tr-H_m_, 6H), 7.40–7.43 (m, Tr-H_o_, 6H); δ (D-threo-**16**) 0.01 (s, CH_3_, 3H), 0.02 (s, CH_3_, 3H), 0.79 (s, tBu, 9H), 0.80 (t, H-6, *J* = 7.1 Hz, 3H), 1.16–1.30 (m, H-4a, H-5, 3H), 1.33–1.38 (m, H-4b, 1H), 2.15 (s, C(O)CH_3_, 3H), 3.17 (dd, H-1a, *J* = 6.8, 10.2 Hz, 1H), 3.25 (dd, H-1b, *J* = 2.7, 10.2 Hz, 1H), 3.86 (ddd, H-3, *J* = 4.3, 5.5, 6.8 Hz, 1H), 5.07 (ddd, H-2, *J* = 2.7, 5.5, 6.8 Hz, 1H), 7.20–7.24 (m, Tr-H_p_, 3H), 7.27-7.30 (m, Tr-H_m_, 6H), 7.40–7.43 (m, Tr-H_o_, 6H); ^13^C NMR (150 MHz, CDCl_3_) δ (l-erythro-**16**) −4.6 (CH_3_), −4.4 (CH_3_), 14.3 (C-6), 18.1 (tBu), 18.7 (C-5), 21.4 (C(O)CH_3_), 25.9 (tBu), 36.1 (C-4), 62.3 (C-1), 72.5 (C-3), 76.0 (C-2), 86.8 (Tr-C_q_), 127.1 (3 × Tr-C_p_), 127.9 (6 × Tr-C_m_), 128.8 (6 × Tr-C_o_), 144.1 (3 × Tr-C_ipso_), 170.5 (C(O)CH_3_); δ (D-threo-**16**) −4.4 (CH_3_), −4.4 (CH_3_), 14.3 (C-6), 18.1 (tBu), 18.5 (C-5), 21.5 (C(O)CH_3_), 25.9 (tBu), 35.1 (C-4), 62.6 (C-1), 70.9 (C-3), 75.8 (C-2), 86.5 (Tr-C_q_), 127.1 (3 × Tr-C_p_), 127.9 (6 × Tr-C_m_), 128.8 (6 × Tr-C_o_), 144.1 (3 × Tr-C_ipso_), 170.7 (C(O)CH_3_); HRMS (ESI) calcd for C_33_H_44_O_4_SiNa^+^ [M + Na]^+^: 555.2901, found: 555.2901.

### 3.7. (2R,3S)-2-O-Acetyl-3-O-tert-butyldimethylsilyl-hexane-1,2,3-triol (l-erythro-17) and (2R,3R)-2-O-acetyl-3-O-tert-butyldimethylsilyl-hexane-1,2,3-triol (d-threo-17)

Formic acid (12.2 mL) was added to a solution of protected triol **16** (959 mg, 1.80 mmol) in diethyl ether (12.2 mL) at 0 °C. The reaction mixture was stirred for 50 min at room temperature, diluted with diethyl ether (30 mL), and cooled to 0 °C. Sat. aqueous NaHCO_3_ (equimolar to formic acid, 323 mmol) was added with vigorous stirring to neutralize the reaction mixture (accompanied by the separation of two layers). The water phase was extracted with diethyl ether (3 × 50 mL) and the combined organic layers were dried and concentrated. The residue was purified by MPLC (gradient AcOEt/hexanes 0/100 to 10/90) to afford 346 mg (66%) of yellowish oil as an inseparable mixture of **17** (l-erythro-**17**/d-threo-**17** 63:37) and **20** (l-erythro-**20**/d-threo-**20** 58:42, the product of the acetyl group migration of **17** taking place even during MPLC) in ratio 82:18. ^1^H NMR (600 MHz, CDCl_3_) δ (l-erythro-**17**) 0.07 (s, CH_3_, 3H), 0.11 (s, CH_3_, 3H), 0.90 (s, tBu, 9H), 0.93 (t, H-6, *J* = 7.3 Hz, 3H), 1.31–1.36 (m, H-5a, 1H), 1.48–1.55 (m, H-4, H-5b, 3H), 2.12 (s, C(O)CH_3_, 3H), 2.68 (dd, OH, *J* = 3.7, 7.7 Hz, 1H), 3.81–3.85 (m, H-1a, 1H), 3.90–3.95 (m, H-1b, H-3, 2H), 4.77 (dt, H-2, *J* = 3.1, 4.9 Hz, 1H); δ (d-threo-**17**) 0.08 (s, CH_3_, 3H), 0.12 (s, CH_3_, 3H), 0.90 (s, tBu, 9H), 0.91 (t, H-6, *J* = 7.2 Hz, 3H), 1.24–1.33 (m, H-5a, 1H), 1.35–1.45 (m, H-4a, H-5b, 2H), 1.46–1.54 (m, H-4b, 1H), 2.11 (s, C(O)CH_3_, 3H), 2.13–2.16 (m, OH, 1H), 3.69–3.73 (m, H-1a, 1H), 3.84–3.90 (m, H-1b, H-3, 2H), 4.87 (ddd, H-2, *J* = 4.1, 4.8, 6.5 Hz, 1H); δ (l-erythro-**20**) 0.08 (s, CH_3_, 3H), 0.09 (s, CH_3_, 3H), 0.90 (s, tBu, 9H), 0.92 (t, H-6, *J* = 7.1 Hz, 3H), 1.29–1.34 (m, H-4a, 1H), 1.38–1.44 (m, H-5, 2H), 1.55–1.61 (m, H-4b, 1H), 2.10 (s, C(O)CH_3_, 3H), 2.32 (d, OH, *J* = 4.7 Hz, 1H), 3.75-3.78 (m, H-3, 1H), 3.78-3.82 (m, H-2, 1H), 4.06 (dd, H-1a, *J* = 7.7, 11.6 Hz, 1H), 4.23 (dd, H-1b, *J* = 3.0, 11.6 Hz, 1H); δ (d-threo-**20**) 0.08 (s, CH_3_, 3H), 0.10 (s, CH_3_, 3H), 0.90 (s, tBu, 9H), 0.92 (t, H-6, *J* = 7.1 Hz, 3H), 1.31-1.44 (m, H-4a, H-5, 3H), 1.64–1.71 (m, H-4a, 1H), 2.09 (s, C(O)CH_3_, 3H), 2.40 (d, OH, *J* = 7.8 Hz, 1H), 3.68-3.71 (m, H-3, 1H), 3.72-3.75 (m, H-2, 1H), 4.05 (dd, H-1a, *J* = 5.1, 11.3 Hz, 1H), 4.08 (dd, H-1b, *J* = 7.0, 11.3 Hz, 1H); ^13^C NMR (150 MHz, CDCl_3_) δ (l-erythro-**17**) −4.5, −4.5 (2 × CH_3_), 14.3 (C-6), 18.2 (tBu), 18.8 (C-5), 21.4 (C(O)CH_3_), 25.9 (tBu), 36.8 (C-4), 61.9 (C-1), 73.8 (C-3), 76.6 (C-2), 171.2 (C(O)CH_3_); δ (d-threo-**17**) = −4.4 (CH_3_), −4.4 (CH_3_), 14.4 (C-6), 18.1 (tBu), 18.9 (C-5), 21.3 (C(O)CH_3_), 25.9 (tBu), 34.8 (C-4), 62.0 (C-1), 71.5 (C-3), 76.7 (C-2), 171.2 (C(O)CH_3_); δ (l-erythro-**20**) −4.5 (CH_3_), −4.3 (CH_3_), 14.4 (C-6), 18.2 (tBu), 18.4 (C-5), 21.1 (C(O)CH_3_), 26.0 (tBu), 34.9 (C-4), 65.8 (C-1), 72.6 (C-2), 73.2 (C-3), 171.5 (C(O)CH_3_); δ (d-threo-**20**) −4.7 (CH_3_),-4.1 (CH_3_), 14.3 (C-6), 18.2 (tBu), 18.6 (C-5), 21.1 (C(O)CH_3_), 26.0 (tBu), 35.9 (C-4), 66.2 (C-1), 70.8 (C-2), 71.9 (C-3), 171.2 (C(O)CH_3_); HRMS (ESI) calcd for C_14_H_30_O_4_SiNa^+^ [M + Na]^+^: 313.1806, found: 313.1806.

### 3.8. (2R,3S)-2-O-Acetyl-4,5,6-trideoxy-3-O-tert-butyldimethylsilyl- l-erythro-hexose (l-erythro-10) and (2R,3R)-2-O-acetyl-4,5,6-trideoxy-3-O-tert-butyldimethylsilyl- d-threo-hexose (d-threo-10)

Oxalyl chloride (2.0 M in CH_2_Cl_2_, 0.85 mL, 1.70 mmol) was added dropwise to a solution of dimethyl sulfoxide (0.18 mL, 2.56 mmol) in dry CH_2_Cl_2_ (3.9 mL) at -78 °C. After 30 min of stirring at −78 °C, a solution of alcohol **17** (330 mg, 82:18 mixture with **20**, 0.93 mmol of **17**) in dry CH_2_Cl_2_ (1.9 mL) was added dropwise. The reaction mixture was stirred at −78 °C for 30 min and Et_3_N (0.63 mL, 4.54 mmol) was added. After 1 h of stirring at −78 °C, the reaction mixture was allowed to reach room temperature slowly (additional 1 h). Subsequently, the reaction mixture was concentrated, dry diethyl ether was added to the residue, and the mixture was filtered through a short silicagel column. The filtrate was concentrated to give the crude aldehyde **10** which was immediately used in the following step without further purification.

### 3.9. (3R,4S)-3-O-Acetyl-1-(2-acetoxymethyl-3-methoxyphenyl)-4-O-tert-butyldimethylsilyl-hept-1-ene-3,4- diol (18) and (3R,4R)-3-O-acetyl-1-(2-acetoxymethyl-3-methoxyphenyl)-4-O-tert-butyldimethylsilyl-hept-1-ene-3,4-diol (19)

Sulfone **11** (457 mg, 1.14 mmol) in dry DME (10.5 mL) was slowly added to a solution of the crude aldehyde **10** (theor. 0.93 mmol) in dry DME (10.5 mL) and the mixture was cooled to −60 °C. Subsequently, KHMDS (0.5 M in toluene, 3.97 mL, 1.99 mmol) was added dropwise keeping −60 °C and the reaction mixture was allowed to reach room temperature. The reaction mixture was stirred for 40 min, quenched with sat. aqueous NH_4_Cl (20 mL), and diluted with AcOEt (20 mL). The aqueous layer was extracted with AcOEt (3 × 20 mL) and the combined organic layers were dried and concentrated. The residue (**18**/**19** 70:30) was repeatedly purified by MPLC (gradient AcOEt/hexanes 0/100 to 5/95) and preparative TLC to afford **18** (79 mg, 18%), **19** (22 mg, 5%), and the mixture of **18** and **19** (78 mg, 18%) as colorless oils over two steps (41% overall yield).

^1^H NMR (600 MHz, CDCl_3_) δ (**18**) 0.06 (s, CH_3_, 3H), 0.10 (s, CH_3_, 3H), 0.90 (t, H-7, *J* = 7.0 Hz, 3H), 0.91 (s, tBu, 9H), 1.30-1.36 (m, H-6a, 1H), 1.39–1.48 (m, H-5, H-6b, 3H), 2.06 (s, C(O)CH_3_), 3H), 2.09 (s, C(O)CH_3_), 3H), 3.84 (s, OCH_3_, 3H), 3.85–3.88 (m, H-4, 1H), 5.25 (d, CH_2_OAc, *J* = 11.8 Hz, 1H), 5.27 (d, CH_2_OAc, *J* = 11.8 Hz, 1H), 5.33 (ddd, H-3, *J* = 1.0, 2.8, 7.6 Hz, 1H), 6.18 (dd, H-2, *J* = 7.6, 15.9 Hz, 1H), 6.84 (d, H-4′, *J* = 8.2 Hz, 1H), 6.85 (d, H-1, *J* = 15.9 Hz, 1H), 7.11 (d, H-6′, *J* = 7.8 Hz, 1H), 7.30 (t, H-5′, *J* = 8.1 Hz, 1H); ^13^C NMR (150 MHz, CDCl_3_) δ (**18**) −4.4 (CH_3_), −4.2 (CH_3_), 14.3 (C-7), 18.4 (tBu), 18.9 (C-6), 21.1 (C(O)CH_3_), 21.5 (C(O)CH_3_), 26.0 (tBu), 36.3 (C-5), 56.0 (OCH_3_), 57.7 (CH_2_OAc), 73.8 (C-4), 77.9 (C-3), 110.3 (C-4′), 118.9 (C-6′), 121.5 (C-2′), 127.5 (C-2), 130.0 (C-5′), 131.2 (C-1), 139.0 (C-1′), 158.6 (C-3′), 170.2 (C(O)CH_3_), 171.2 (C(O)CH_3_); HRMS (ESI) calcd for C_25_H_40_O_6_SiNa^+^ [M + Na]^+^: 487.2486, found: 487.2486; [α]${}_{D}^{20}$ −48.8 (c 1.131 MeOH).

^1^H NMR (600 MHz, CDCl_3_) δ (**19**) 0.08 (s, CH_3_, 3H), 0.11 (s, CH_3_, 3H), 0.89 (t, H-7, *J* = 7.0 Hz, 3H), 0.90 (s, tBu, 9H), 1.30–1.37 (m, H-6a, 1H), 1.38-1.50 (m, H-5, H-6b, 3H), 2.06 (s, C(O)CH_3_), 3H), 2.11 (s, C(O)CH_3_), 3H), 3.80 (ddd, H-4, *J* = 3.6, 6.0, 7.2 Hz, 1H), 3.84 (s, OCH_3_, 3H), 5.26 (s, CH_2_OAc, 2H), 5.38 (ddd, H-3, *J* = 1.4, 6.1, 6.1 Hz, 1H), 6.11 (dd, H-2, *J* = 6.2, 15.9 Hz, 1H), 6.83 (d, H-4′, *J* = 8.3 Hz, 1H), 6.84 (dd, H-1, *J* = 1.3, 15.9 Hz, 1H), 7.09 (d, H-6′, *J* = 7.8 Hz, 1H), 7.29 (t, H-5′, *J* = 8.1 Hz, 1H); ^13^C NMR (150 MHz, CDCl_3_) δ (**19**) −4.4 (CH_3_), −4.2 (CH_3_), 14.5 (C-7), 18.2 (tBu), 18.5 (C-6), 21.1 (C(O)CH_3_), 21.4 (C(O)CH_3_), 26.0 (tBu), 35.3 (C-5), 56.0 (OCH_3_), 57.7 (CH_2_OAc), 73.0 (C-4), 76.8 (C-3), 110.2 (C-4′), 118.8 (C-6′), 121.4 (C-2′), 128.1 (C-2), 129.5 (C-1), 130.0 (C-5′), 139.0 (C-1′), 158.6 (C-3′), 170.2 (C(O)CH_3_), 171.2 (C(O)CH_3_); HRMS (ESI) calcd for C_25_H_40_O_6_SiNa^+^ [M + Na]^+^: 487.2486, found: 487.2486; [α]${}_{D}^{20}$ +5.5 (c 0.431 MeOH).

### 3.10. Varioxiranol A (4)

Compound **18** (21.1 mg, 0.045 mmol) was dissolved in methanol (2 mL) and K_2_CO_3_ (12.6 mg, 0.091 mol) was added. The reaction mixture was stirred at room temperature for 2.5 h, diluted with AcOEt (4 mL) and with water (4 mL). The water phase was extracted with AcOEt (4 × 2 mL) and combined organic layers were dried and concentrated. The residue was diluted in THF (0.5 mL), the solution was cooled to 0 °C, and TBAF × 3H_2_O in THF (1.0 M solution, 46 µL, 0.046 mmol) was added. The reaction mixture was stirred at room temperature for 4.5 h and quenched with sat. aqueous NH_4_Cl (5 mL), the aqueous layer was extracted with CH_2_Cl_2_ (3 × 5 mL), and the combined organic layers were dried and concentrated. The residue was purified by FLC (isocratic acetone/CH_2_Cl_2_ 20/80 then 50/50) to afford varioxiranol A (**4**, 11.1 mg, 92% over two steps) as colorless crystalline solid that was subsequently recrystallized from AcOEt-hexanes. ^1^H NMR (600 MHz, CDCl_3_) δ (**4**) 0.94 (t, H-7, *J* = 7.2 Hz, 3H), 1.36-1.43 (m, H-6a, 1H), 1.43–1.48 (m, H-5, 2H), 1.51–1.57 (m, H-6b, 1H), 2.09 (d, OH, *J* = 4.3 Hz, 1H), 2.20 (t, OH, *J* = 5.8 Hz, 1H), 2.22 (d, OH, *J* = 4.6 Hz, 1H), 3.78–3.82 (m, H-4, 1H), 3.87 (s, OCH_3_, 3H), 4.26–4.30 (m, H-3, 1H), 4.76–4.83 (m, CH_2_OH, 2H), 6.19 (dd, H-2, *J* = 6.9, 15.8 Hz, 1H), 6.84 (d, H-4′, *J* = 8.2 Hz, 1H), 7.02 (d, H-1, *J* = 15.8 Hz, 1H), 7.10 (d, H-6′, *J* = 7.9 Hz, 1H), 7.25 (t, H-5′, *J* = 8.2 Hz, 1H); ^13^C NMR (150 MHz, acetone-d_6_) δ (**4**) 14.5 (C-7), 19.8 (C-6), 35.7 (C-5), 55.5 (CH_2_OH), 56.1 (OCH_3_), 75.0 (C-4), 76.7 (C-3), 110.4 (C-4′), 119.4 (C-6′), 127.9 (C-2′), 129.2 (C-1), 129.3 (C-5′), 133.7 (C-2), 139.6 (C-1′), 158.9 (C-3′); HRMS (ESI) calcd for C_15_H_22_O_4_Na^+^ [M+Na]^+^: 289.1410, found: 289.1410; [α]${}_{D}^{20}$ +2.5 (c 0.207 MeOH); mp 120–121 °C.

### 3.11. 4-epi-Varioxiranol A (9)

Compound **19** (20.8 mg, 0.045 mmol) was dissolved in methanol (2 mL) and K_2_CO_3_ (12.4 mg, 0.090 mmol) was added. The reaction mixture was stirred at room temperature for 2.5 h and diluted with AcOEt (4 mL) and with water (4 mL). The water phase was extracted with AcOEt (4 × 2 mL) and combined organic layers were dried and concentrated. The residue was diluted in THF (0.5 mL), the solution was cooled to 0 °C, and TBAF × 3H_2_O in THF (1.0 M solution, 45 µL, 0.045 mmol) was added. The reaction mixture was stirred at room temperature for 4.5 h and quenched with sat. aqueous NH_4_Cl (5 mL). The aqueous layer was extracted with CH_2_Cl_2_ (3 × 5 mL) and the combined organic layers were dried and concentrated. The residue was purified by FLC (isocratic acetone/CH_2_Cl_2_ 30/70) to afford 4-epi-varioxiranol A (**9**, 6.8 mg, 57% over two steps) that was subsequently recrystallized from CH_2_Cl_2_-hexanes yielding colorless crystalline solid. ^1^H NMR (600 MHz, CDCl_3_) δ (**9**) 0.94 (t, H-7, *J* = 7.1 Hz, 3H), 1.39–1.45 (m, H-6a, 1H), 1.46–1.56 (m, H-5, H-6b, 3H), 2.20 (t, OH, *J* = 6.3 Hz, 1H), 2.28 (d, OH, *J* = 4.3 Hz, 1H), 2.40 (d, OH, *J* = 4.3 Hz, 1H), 3.56–3.61 (m, H-4, 1H), 3.87 (s, OCH_3_, 3H), 4.10–4.14 (m, H-3, 1H), 4.78 (dd, CH_2_OH, *J* = 6.3, 12.1 Hz, 1H), 4.81 (dd, CH_2_OH, *J* = 6.3, 12.1 Hz, 1H), 6.12 (dd, H-2, *J* = 6.8, 15.8 Hz, 1H), 6.84 (d, H-4′, *J* = 8.2 Hz, 1H), 7.04 (d, H-1, *J* = 15.8 Hz, 1H), 7.08 (d, H-6′, *J* = 7.8 Hz, 1H), 7.25 (t, H-5′, *J* = 8.0 Hz, 1H); ^13^C NMR (150 MHz, CDCl_3_) δ (**9**) 14.2 (C-7), 19.0 (C-6), 35.4 (C-5), 55.8 (OCH_3_), 56.6 (CH_2_OH), 74.5 (C-4), 76.3 (C-3), 110.0 (C-4′), 119.4 (C-6′), 126.4 (C-2′), 129.1 (C-5′), 129.8 (C-1), 132.6 (C-2), 137.7 (C-1′), 158.2 (C-3′); HRMS (ESI) calcd for C_15_H_22_O_4_Na^+^ [M + Na]^+^: 289.1410, found: 289.1410; [α]${}_{D}^{20}$ +45.0 (c 0.094 MeOH); mp 112–113 °C.

## Supplementary Materials

The following are available online, S1: Copies of ^1^H NMR and ^13^C NMR spectra for all new compounds.
