# Supplementary material for: First Total Synthesis of Varioxiranol A
Source: Molecules. 2019 Feb 28;24(5):862. doi: 10.3390/molecules24050862 (PMC6429112; doi:10.3390/molecules24050862)

Supporting information

First total synthesis of natural varioxiranol A

Angelika Lásiková[1,*], Jana Doháňošová[2], Mária Štiblariková[1], Martin Parák[1], Ján Moncol[3] and Tibor Gracza[1]

[1] Department of Organic Chemistry, Slovak University of Technology, Radlinského 9, 812 37 Bratislava, Slovakia; angelika.lasikova@stuba.sk; maria.stiblarikova@stuba.sk; tibor.gracza@stuba.sk

[2] Central Laboratories, Slovak University of Technology, Radlinského 9, 812 37 Bratislava, Slovakia; jana.dodanosova@stuba.sk

[3] Department of Inorganic Chemistry, Slovak University of Technology, Radlinského 9, 812 37 Bratislava, Slovakia; jan.moncol@stuba.sk

* Correspondence: angelika.lasikova@stuba.sk; Tel.: +421-2-593-25-167

Table of contents

1. ^{1}H and ^{13}C NMR spectra of compounds *L-erythro*-**12** and *D-threo*-**12** S2
2. ^{1}H and ^{13}C NMR spectra of compounds *L-erythro*-**13** and *D-threo*-**13** S3
3. ^{1}H and ^{13}C NMR spectra of compounds *L-erythro*-**14** and *D-threo*-**13** S4
4. ^{1}H and ^{13}C NMR spectra of compounds *L-erythro*-**15** and *D-threo*-**15** S5
5. ^{1}H and ^{13}C NMR spectra of compounds *L-erythro*-**16** and *D-threo*-**16** S6
6. ^{1}H and ^{13}C NMR spectra of compounds *L-erythro*-**17** and *D-threo*-**17** S7
7. ^{1}H and ^{13}C NMR spectra of compound **18** S8
8. ^{1}H and ^{13}C NMR spectra of compound **19** S9
9. ^{1}H and ^{13}C NMR spectra of varioxiranol A (**4**) S10
10. ^{1}H and ^{13}C NMR spectra of 4-*epi*-varioxiranol A (**9**) S11
11. Table S1 Crystal data and structure refinement for compounds **4** and **9** S12
12. Table S2 Hydrogen bonds parameters of **4** and **9** S13
13. ORTEP-like drawing of **4** S14
14. ORTEP-like drawing of **9** S15
15. Hydrogen bond network in crystal structure of **4** S16
16. Hydrogen bond network in crystal structure of **9** S17

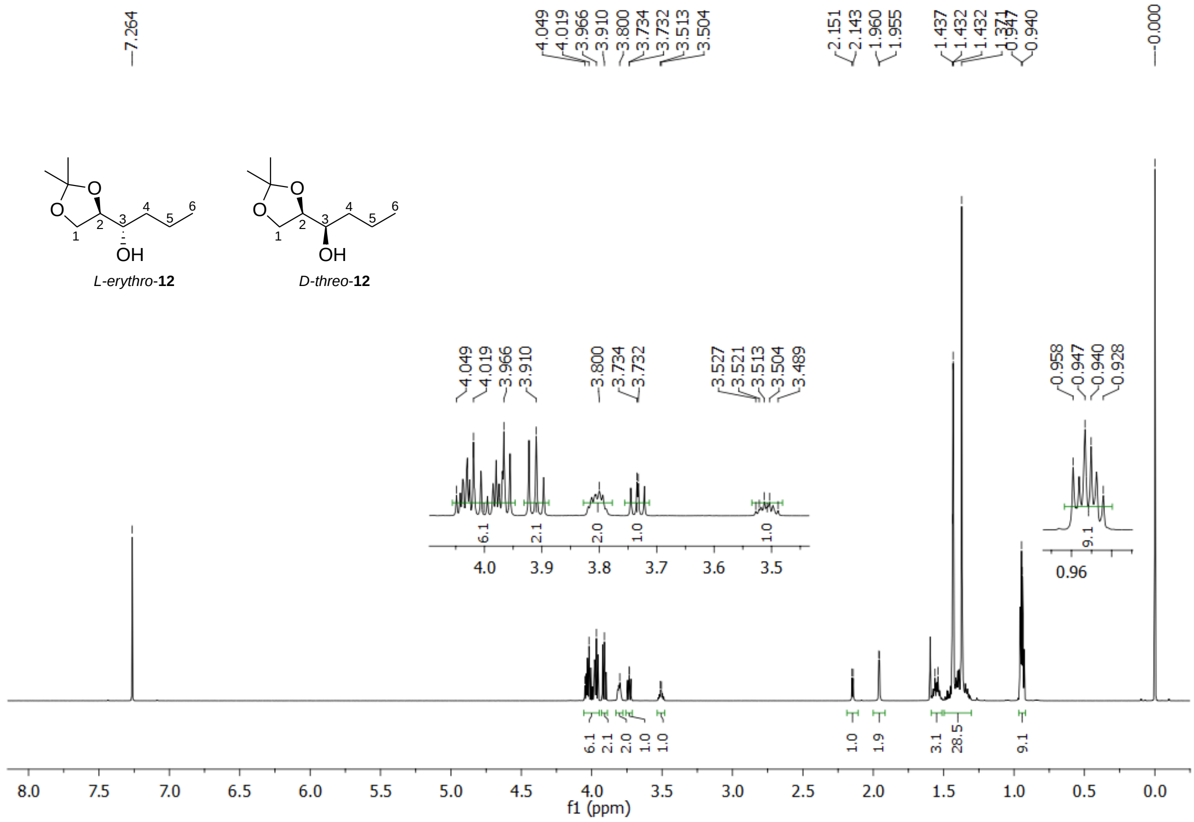
7.264
4.049
4.019
3.966
3.910
3.800
3.734
3.732
3.513
3.504
2.151
2.143
1.960
1.955
1.437
1.432
1.432
0.940
0.000
L-erythro-12
D-threo-12
4.049
4.019
3.966
3.910
3.800
3.734
3.732
3.527
3.521
3.513
3.504
3.489
0.958
0.947
0.940
0.928
6.1
2.1
2.0
1.0
1.0
9.1
0.96
1.0
1.9
3.1
28.5
f1 (ppm)

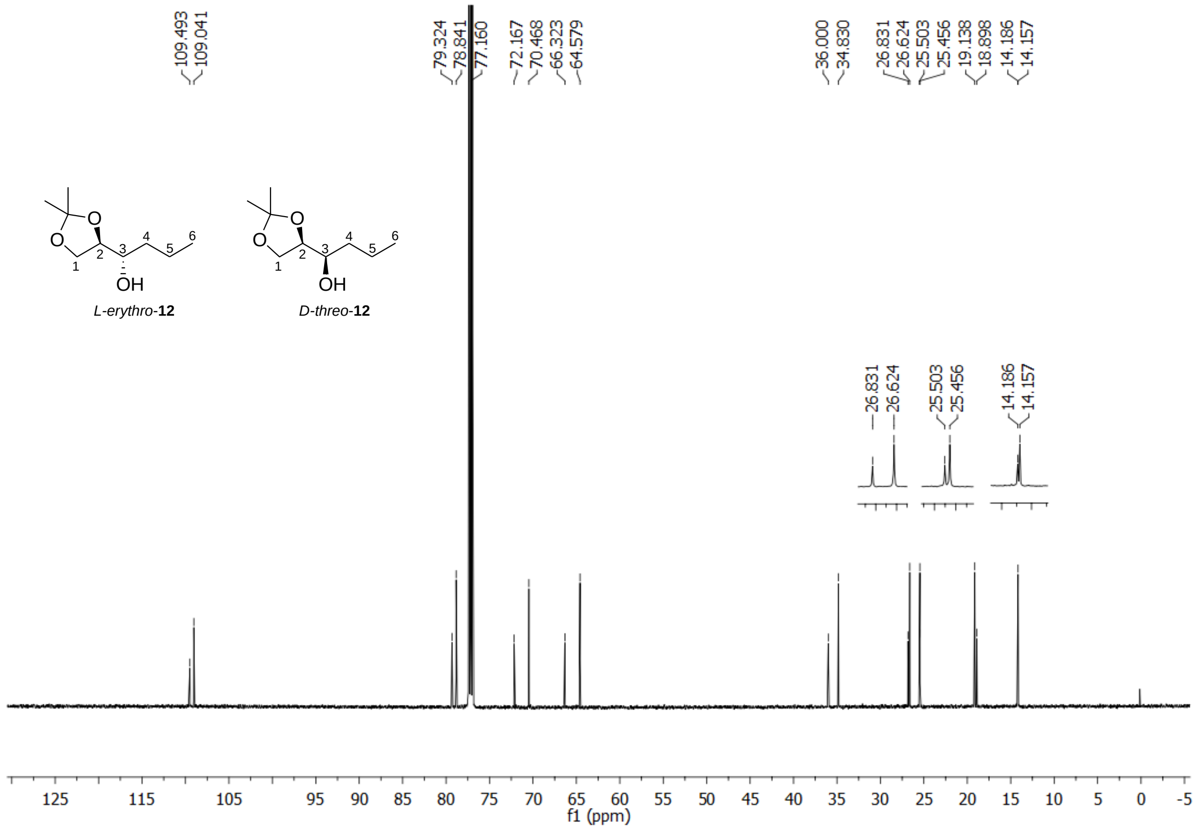
109.493
109.041
79.324
78.841
77.160
72.167
70.468
66.323
64.579
36.000
34.830
26.831
26.624
25.503
25.456
19.138
18.898
14.186
14.157
L-erythro-12
D-threo-12
f1 (ppm)

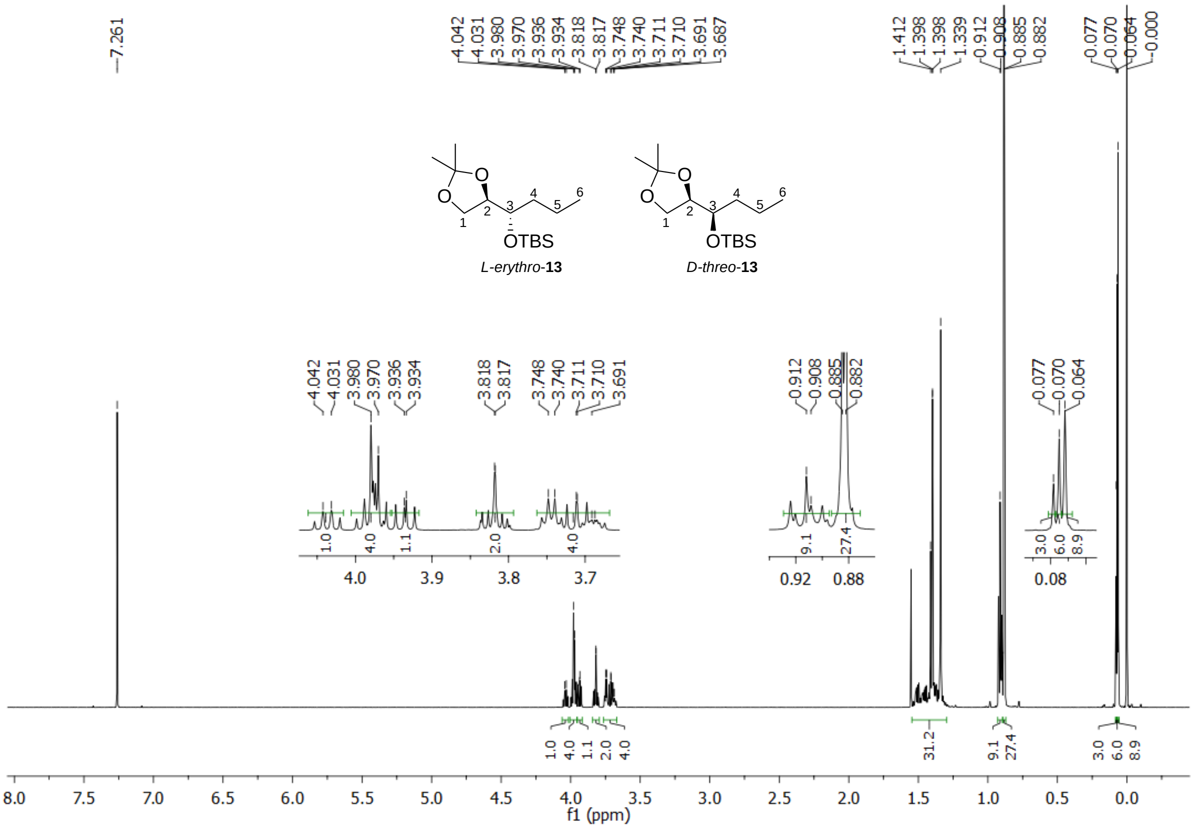

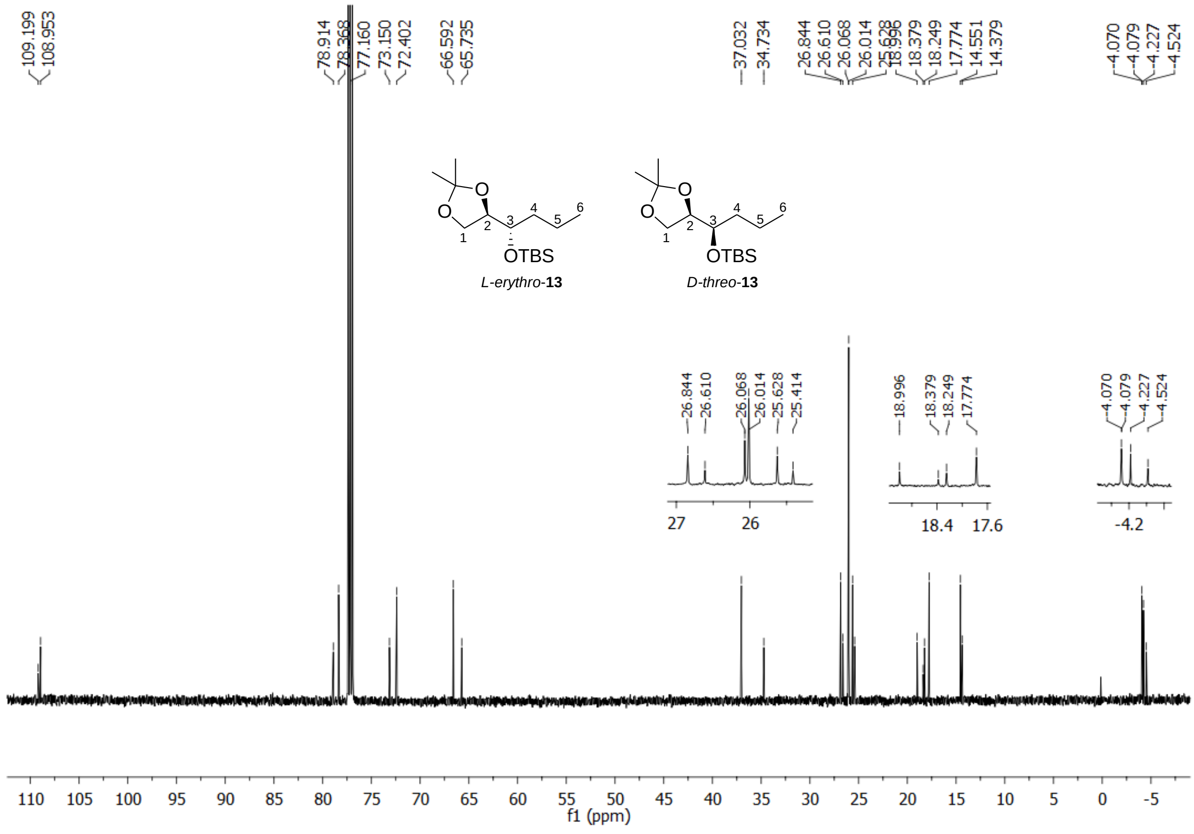

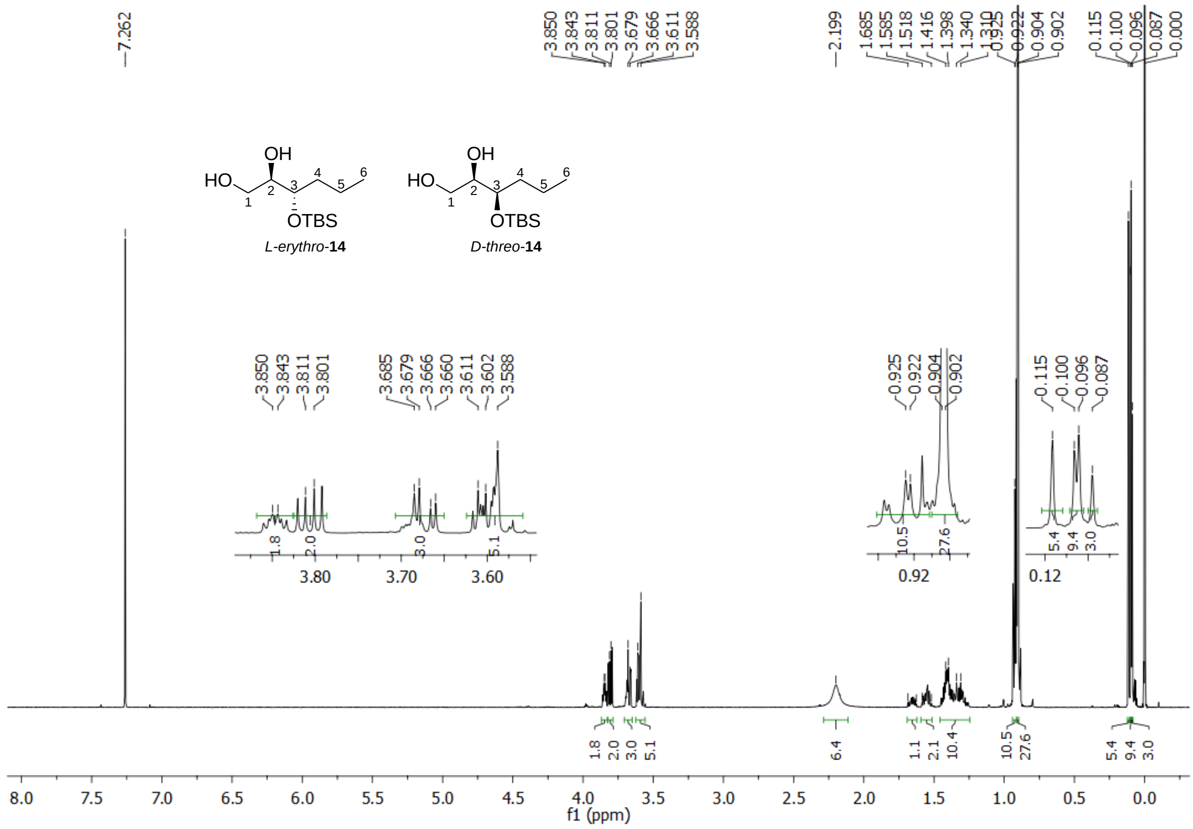
L-erythro-14
D-threo-14
f1 (ppm)

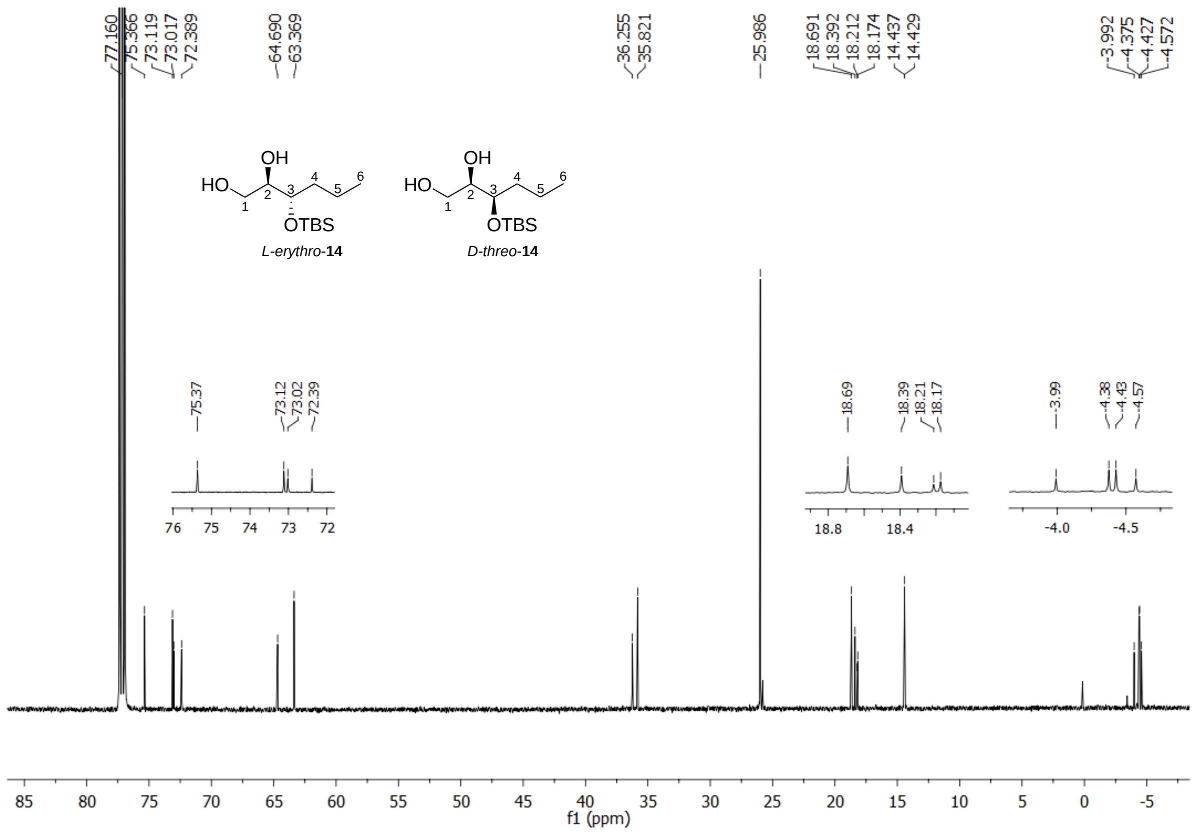
L-erythro-14
D-threo-14
f1 (ppm)

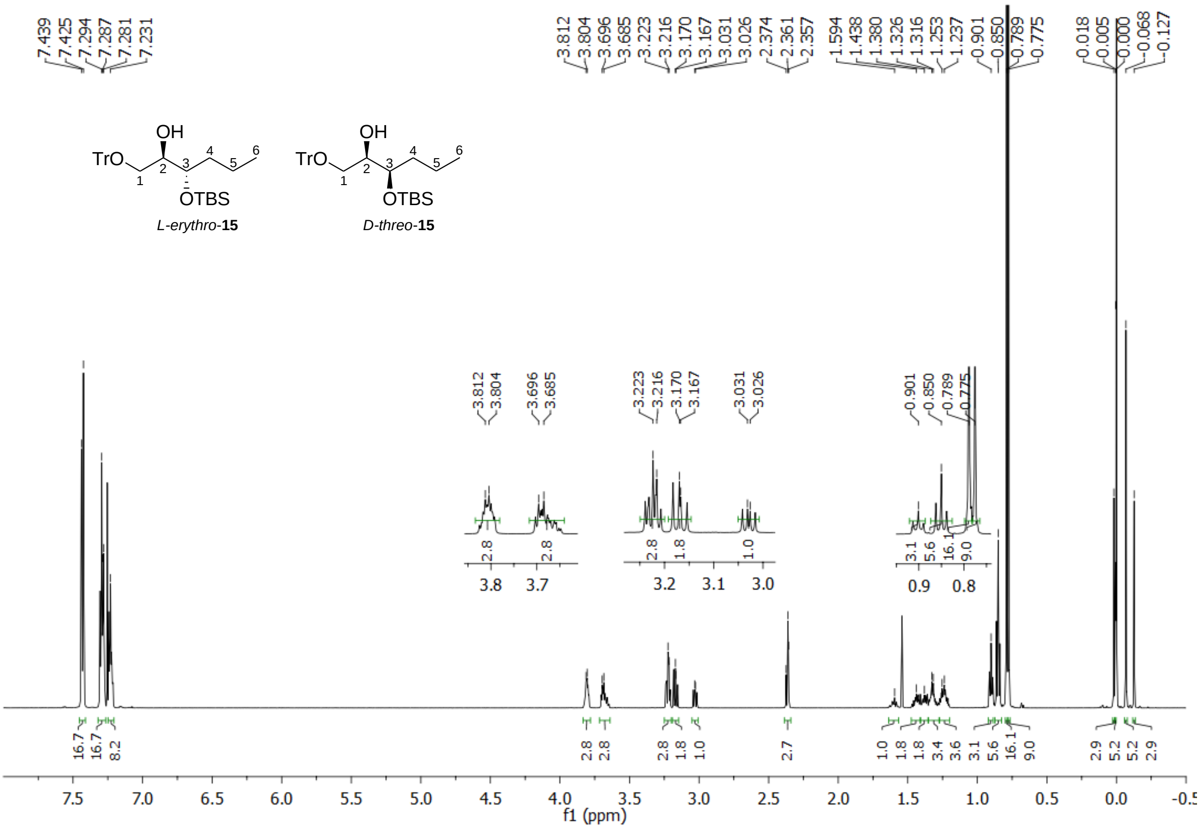
7.439 7.425 7.294 7.287 7.281 7.231
3.812 3.804 3.696 3.685 3.223 3.216 3.170 3.167 3.031 3.026 2.374 2.361 2.357
1.594 1.438 1.380 1.326 1.316 1.253 1.237 0.901 0.850 0.789 0.775
0.018 0.005 0.000 -0.068 -0.127
TrO OH OTBS
L-erythro-15
D-threo-15
f1 (ppm)

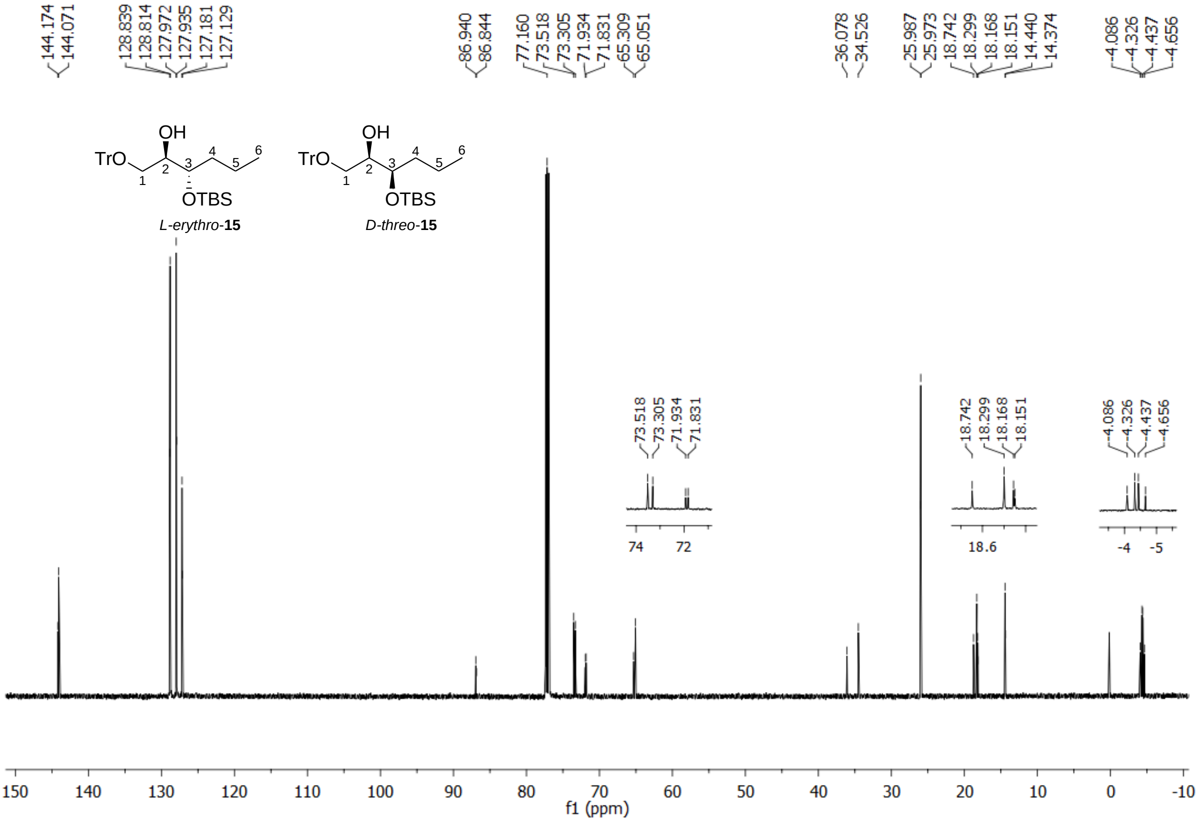
144.174 144.071
128.839 128.814 127.972 127.935 127.181 127.129
86.940 86.844
77.160 73.518 73.305 71.934 71.831 65.309 65.051
36.078 34.526
25.987 25.973 18.742 18.299 18.168 18.151 14.440 14.374
-4.086 -4.326 -4.437 -4.656
TrO OH OTBS
L-erythro-15
D-threo-15
f1 (ppm)

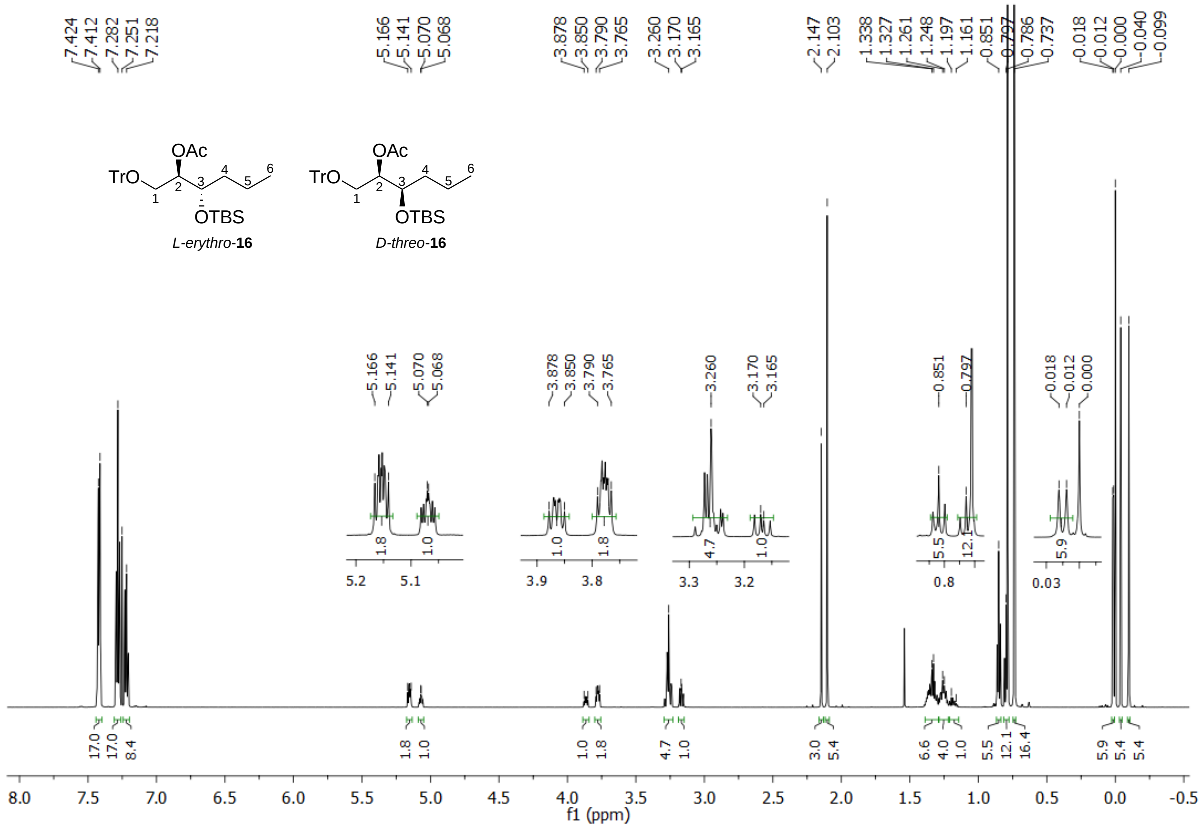

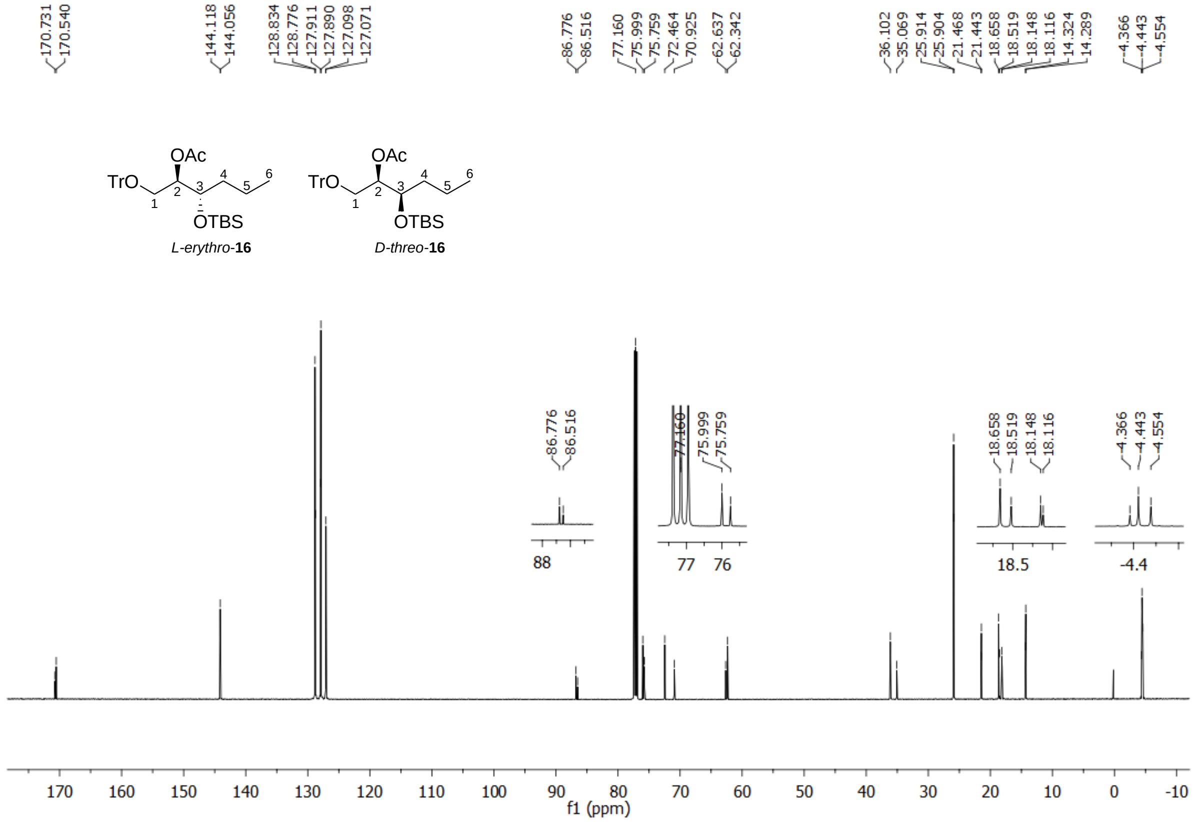

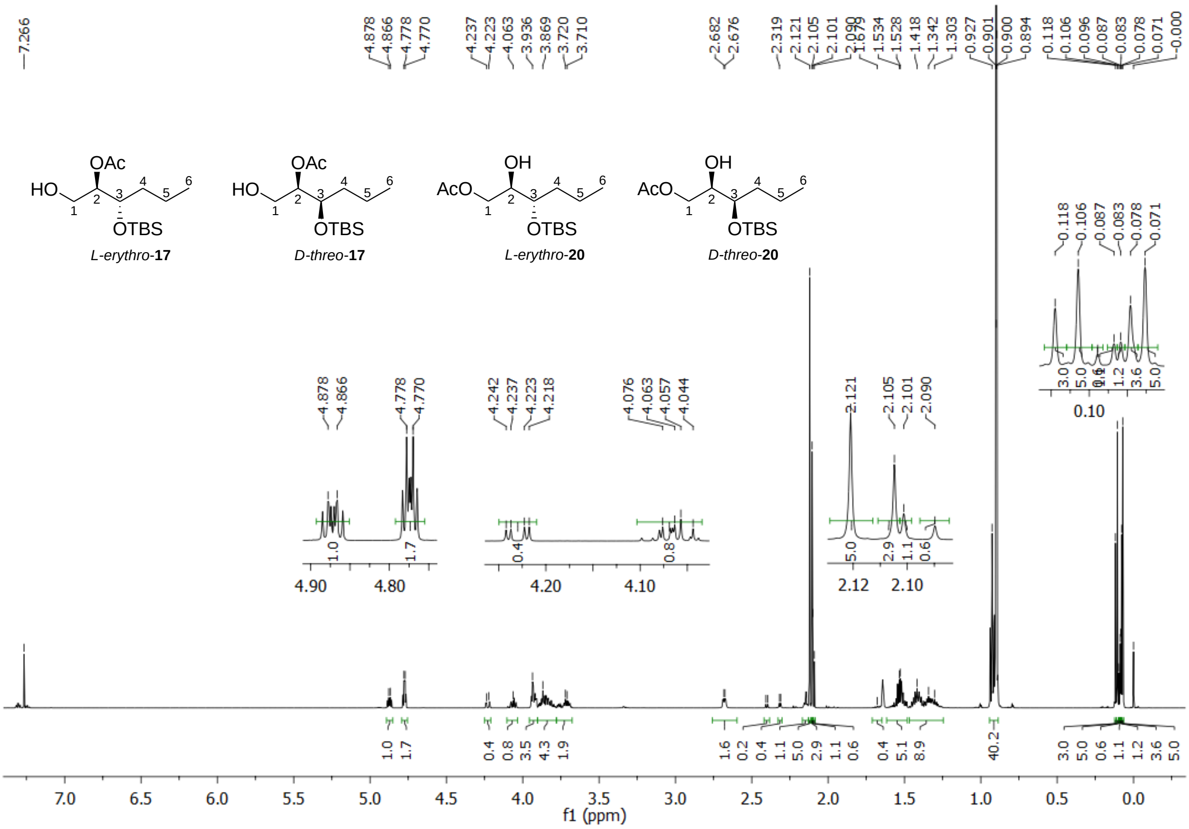
L-erythro-17
D-threo-17
L-erythro-20
D-threo-20
f1 (ppm)

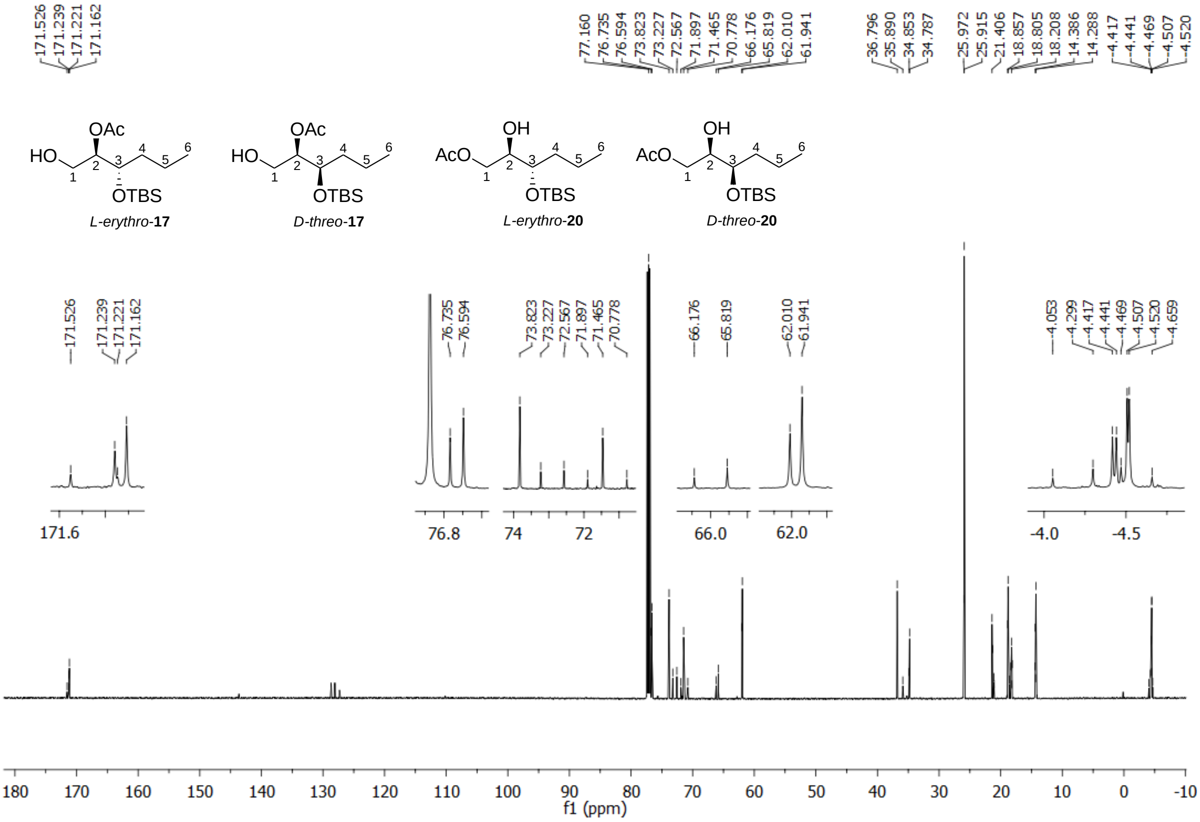
L-erythro-17
D-threo-17
L-erythro-20
D-threo-20
f1 (ppm)

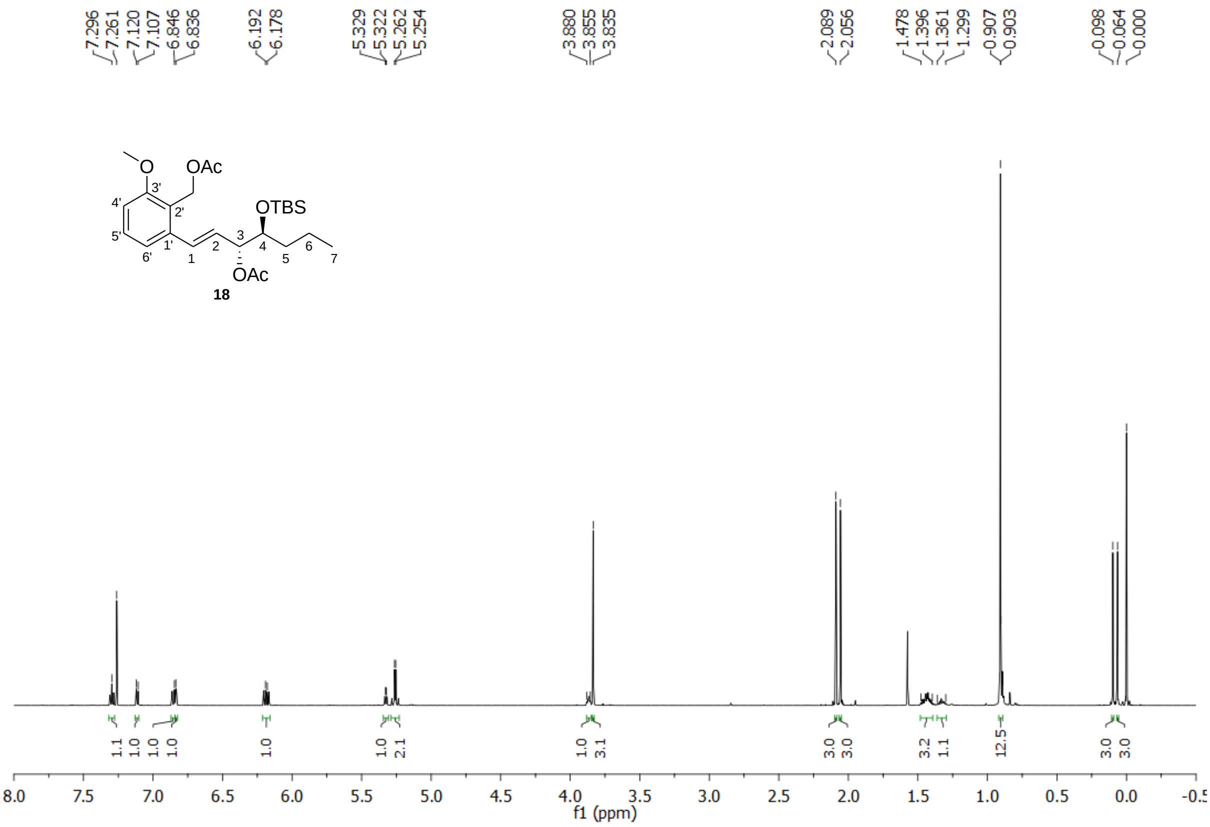
7.296
7.261
7.120
7.107
6.846
6.836
6.192
6.178
5.329
5.322
5.262
5.254
3.880
3.855
3.835
2.089
2.056
1.478
1.396
1.361
1.299
0.907
0.903
0.098
0.064
0.000
OAc
OTBS
OAc
3'
2'
4'
5'
6'
1'
1
2
3
4
5
6
7
18
1.1
1.0
1.0
1.0
1.0
1.0
2.1
1.0
3.1
3.0
3.0
3.2
1.1
12.5
3.0
3.0
8.0 7.5 7.0 6.5 6.0 5.5 5.0 4.5 4.0 3.5 3.0 2.5 2.0 1.5 1.0 0.5 0.0
f1 (ppm)

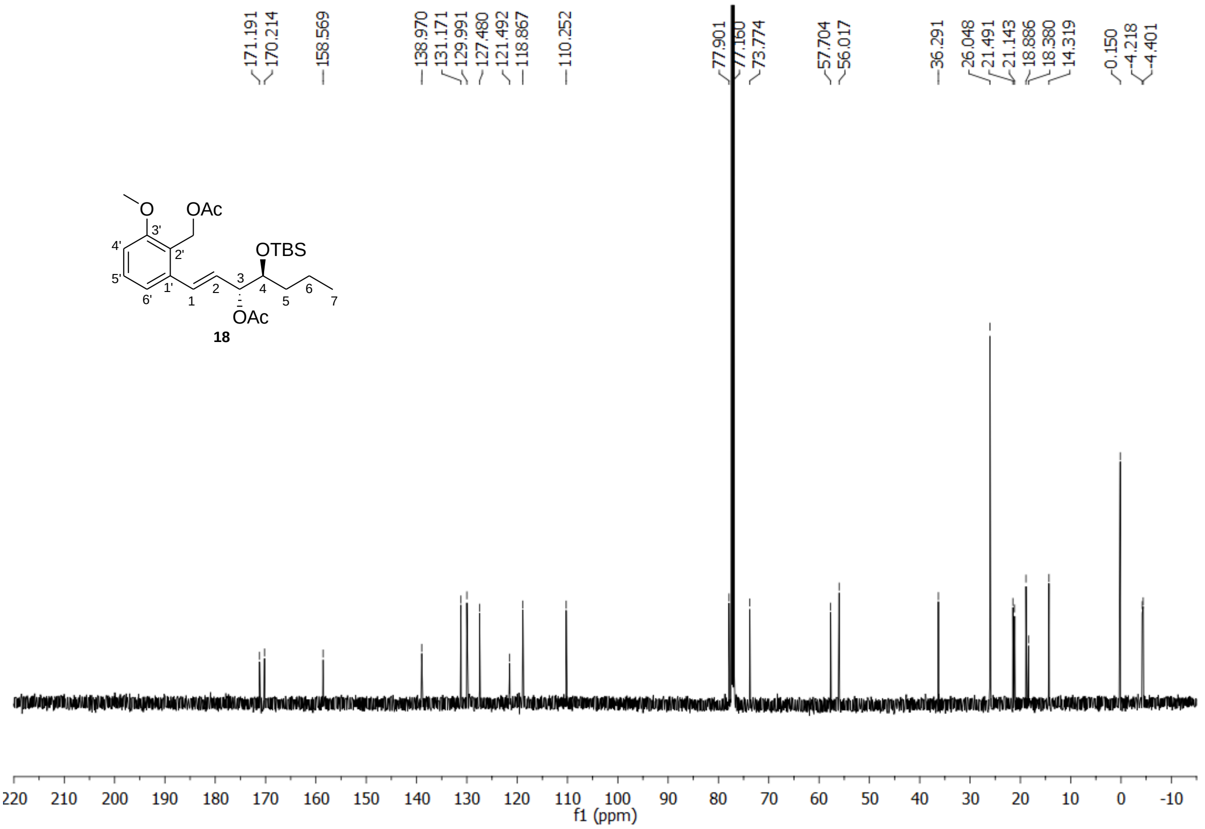
171.191
170.214
158.569
138.970
131.171
129.991
127.480
121.492
118.867
110.252
77.901
77.160
73.774
57.704
56.017
36.291
26.048
21.491
21.143
18.886
18.380
14.319
-0.150
-4.218
-4.401
OAc
OTBS
OAc
18
220 210 200 190 180 170 160 150 140 130 120 110 100 90 80 70 60 50 40 30 20 10 0 -10
f1 (ppm)

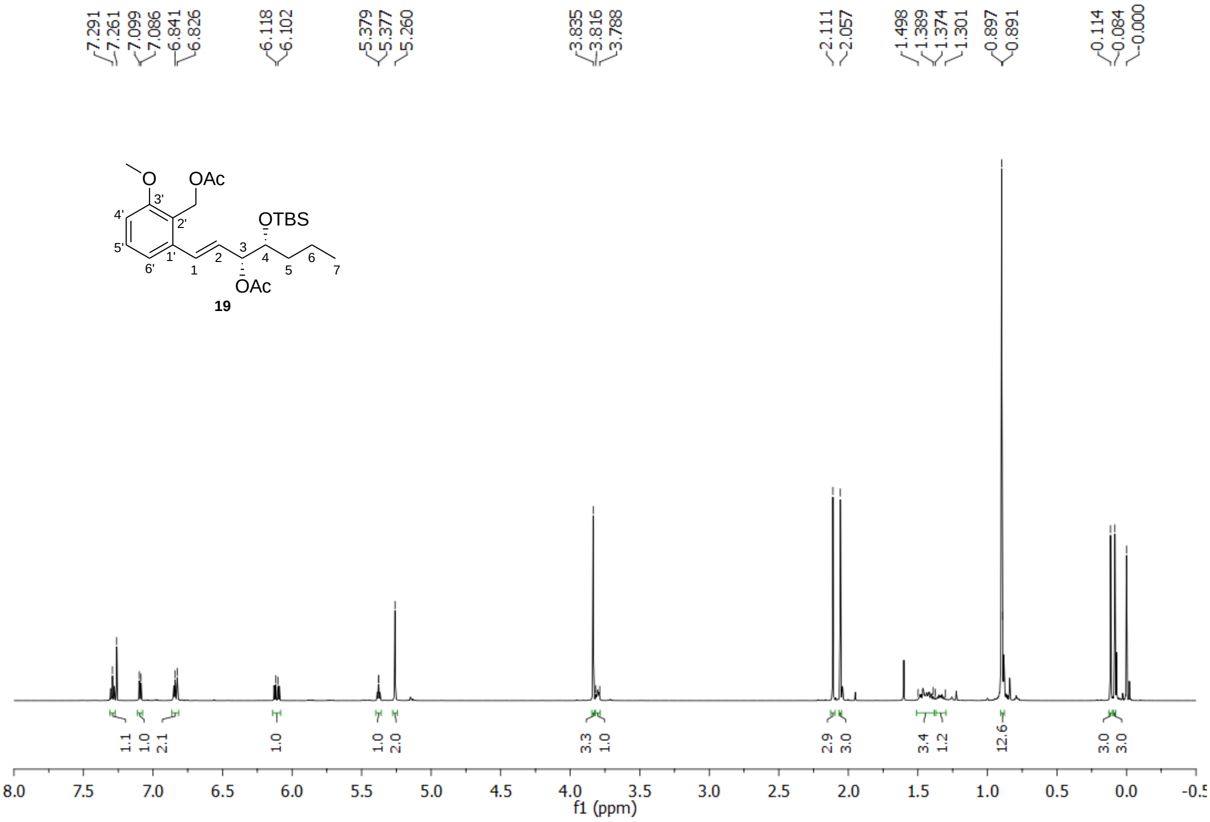
7.291
7.261
7.099
7.086
6.841
6.826
6.118
6.102
5.379
5.377
5.260
3.835
3.816
3.788
2.111
2.057
1.498
1.389
1.374
1.301
0.897
0.891
0.114
0.084
-0.000
OAc
OTBS
OAc
19
1.1
1.0
2.1
1.0
1.0
2.0
3.3
1.0
2.9
3.0
3.4
1.2
12.6
3.0
3.0
f1 (ppm)

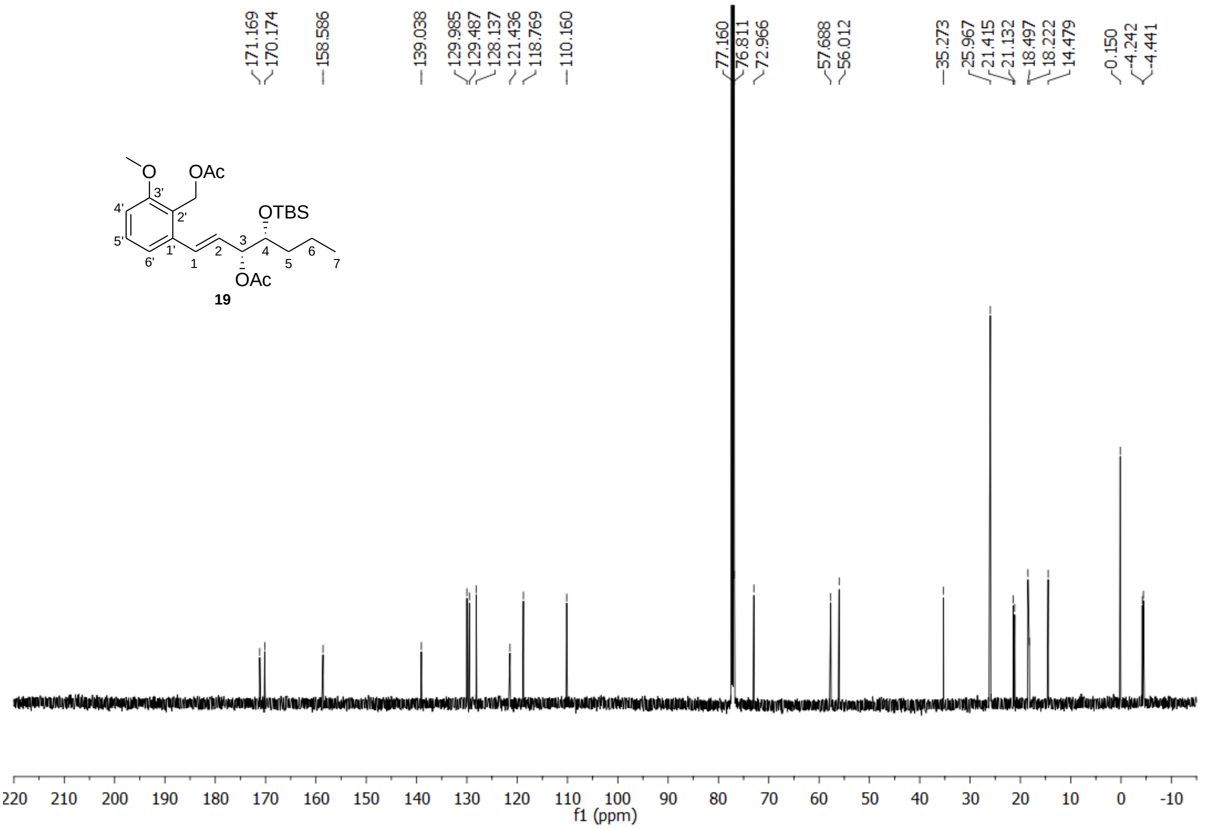
171.169
170.174
158.586
139.038
129.985
129.487
128.137
121.436
118.769
110.160
77.160
76.811
72.966
57.688
56.012
35.273
25.967
21.415
21.132
18.497
18.222
14.479
-0.150
-4.242
-4.441
OAc
OTBS
OAc
19
f1 (ppm)

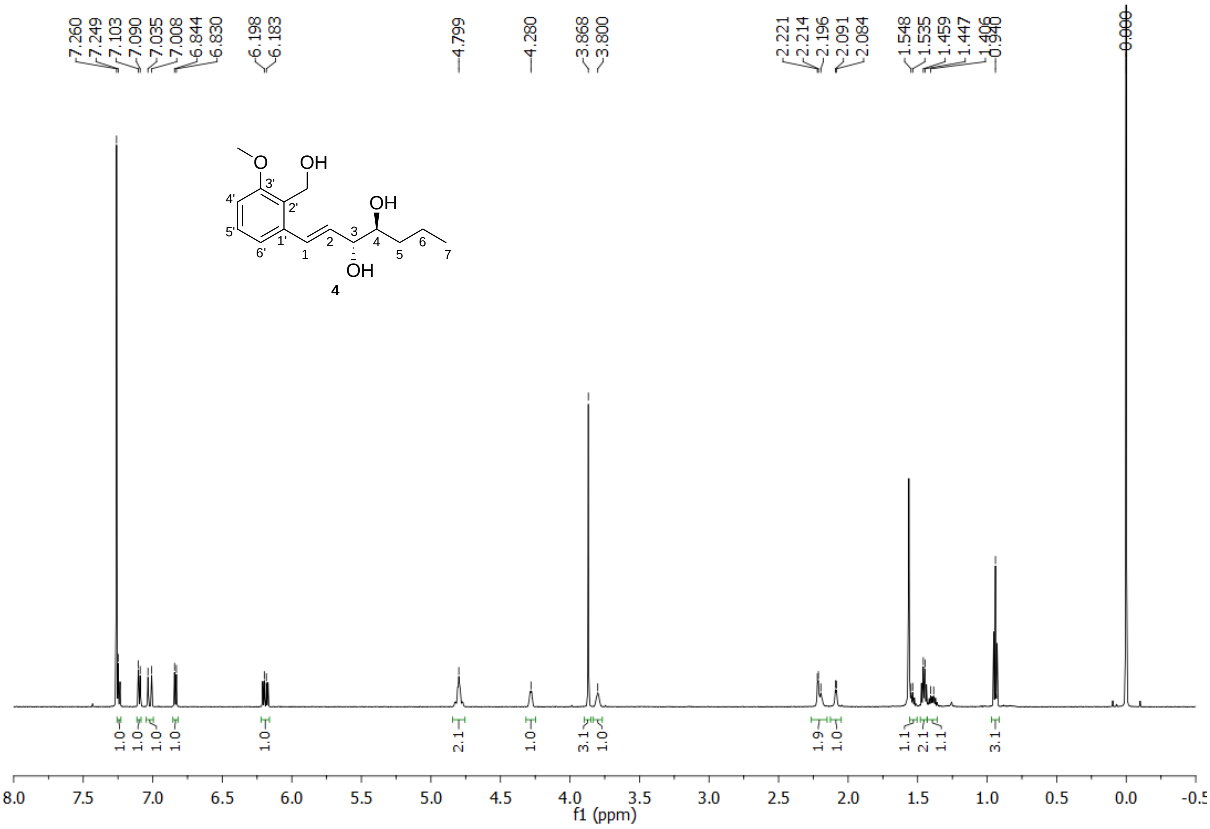
7.260
7.249
7.103
7.090
7.035
7.008
6.844
6.830
6.198
6.183
4.799
4.280
3.868
3.800
2.221
2.214
2.196
2.091
2.084
1.548
1.535
1.459
1.447
0.940
0.000
OH
OH
OH
O
1
2
3
4
5
6
7
1'
2'
3'
4'
5'
6'
4
f1 (ppm)

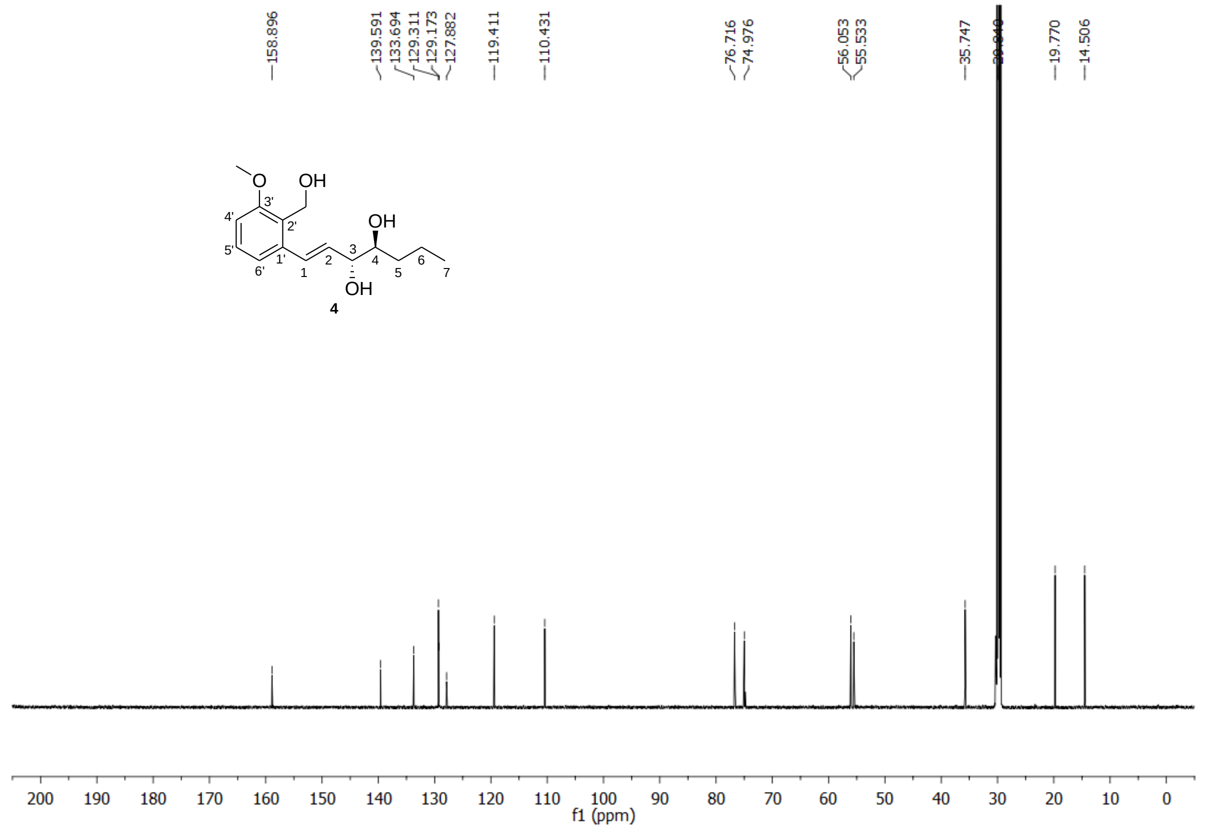
158.896
139.591
133.694
129.311
129.173
127.882
119.411
110.431
76.716
74.976
56.053
55.533
35.747
19.770
14.506
OH
OH
OH
O
4
f1 (ppm)

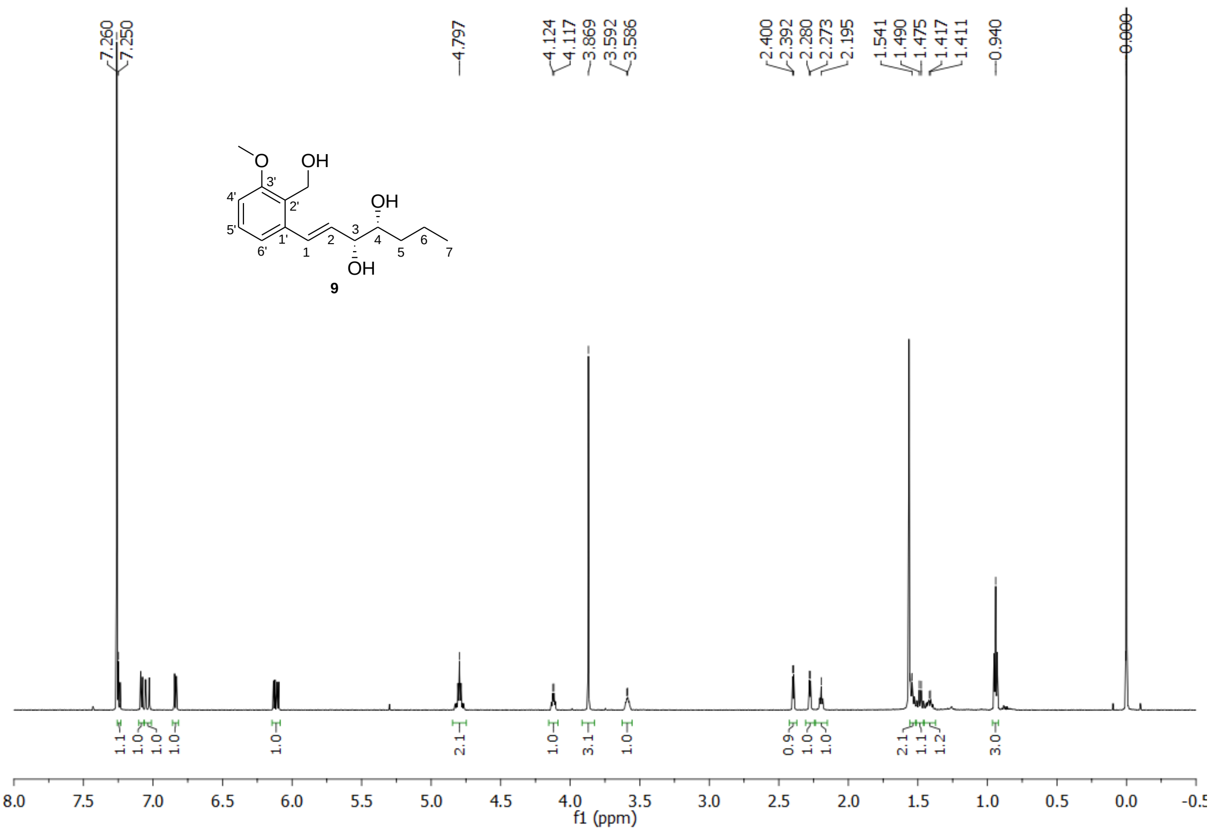
7.260
7.250
4.797
4.124
4.117
3.869
3.592
3.586
2.400
2.392
2.280
2.273
2.195
1.541
1.490
1.475
1.417
1.411
0.940
0.000
OH
OH
OH
9
1.1
1.0
1.0
1.0
1.0
2.1
1.0
3.1
1.0
0.9
1.0
1.0
2.1
1.1
1.2
3.0
f1 (ppm)

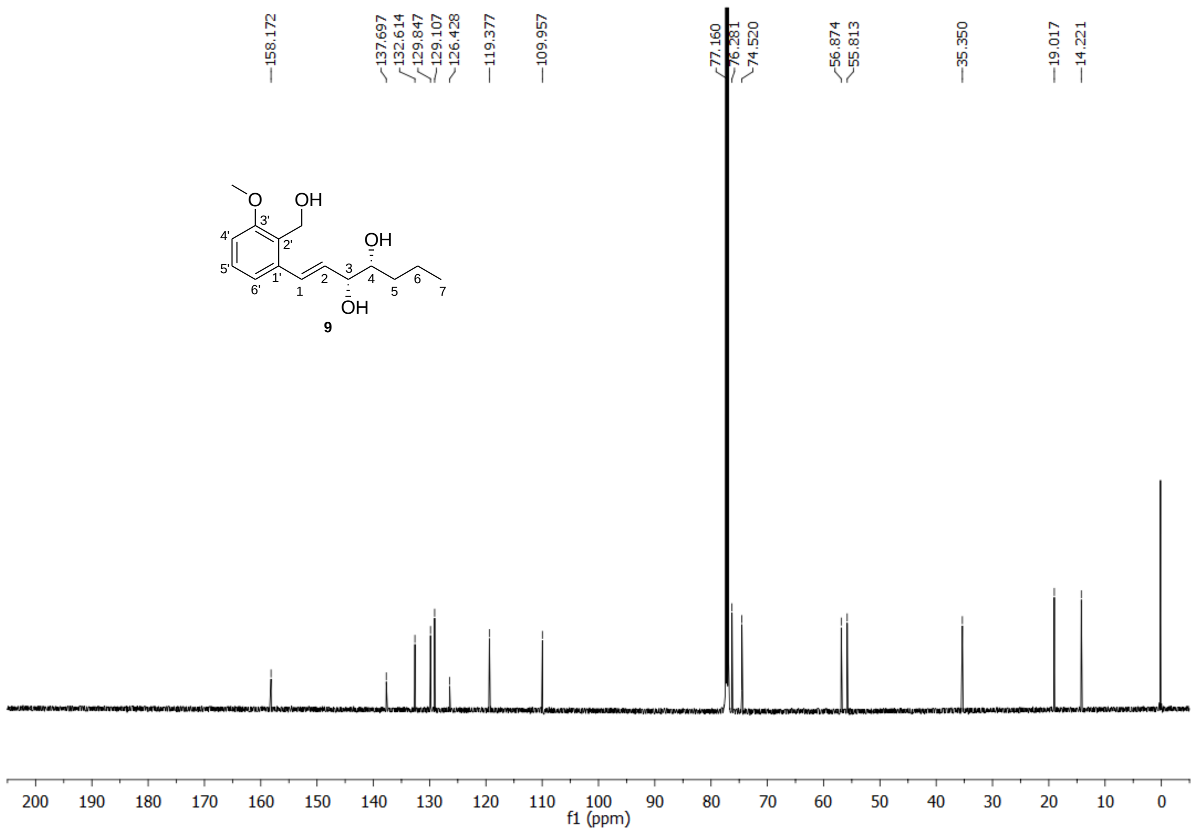
158.172
137.697
132.614
129.847
129.107
126.428
119.377
109.957
77.160
76.281
74.520
56.874
55.813
35.350
19.017
14.221
OH
OH
OH
9
f1 (ppm)

Table S1 Crystal data and structure refinement for compounds **4** and **9**

	**4**	**9**
Empirical formula	$C_{15}H_{22}O_4$	$C_{15}H_{22}O_4$
Formula weight /g mol^{-1}	266.32	266.32
Crystal system	monoclinic	monoclinic
Space group	$P2_1$	$P2_1$
Temperature /K	100	100
Crystal size /mm	0.24 × 0.03 × 0.02	0.42 × 0.07 × 0.02
Z	2	4
a / Å	11.3505(4)	12.4108(1)
b / Å	4.9503(2)	5.0577(1)
c / Å	13.0003(6)	22.8288(2)
β /°	104.764(4)	101.593(2)
V /Å^3	706.35(5)	1403.73(1)
ρ_{calc} /g cm^{-3}	1.252	1.260
μ /mm^{-1}	0.730	0.735
F(000)	288.0	576.0
Radiation	CuKα , (λ = 1.54186 Å)	CuKα, (λ = 1.54186 Å)
2Θ range for data collection/°	7.032 to 145.314	7.270 to 143.468
Index ranges	-11 ≤ h ≤ 14,	-15 ≤ h ≤ 14,
	-5 ≤ k ≤ 6,	-3 ≤ k ≤ 6,
	-16 ≤ l ≤ 10	-25 ≤ l ≤ 28
Data/restraints/parameters	2407/1/178	4184/13/368
Goodness-of-fit on F^2	1.038	0.987
Final R indexes [I>=2σ (I)]	R_1 = 0.0459, wR_2 = 0.1206	R_1 = 0.0317, wR_2 = 0.0722
R indices (all data)	R_1 = 0.0534, wR_2 = 0.1272	R_1 = 0.0367, wR_2 = 0.0755
Flack parameter (x)	0.3(2)	-0.08(5)
Hooft parameter (y)	0.07(11)	-0.02(5)
CCDC No.	1892452	1892453

Table S2 Hydrogen bonds parameters of **4** and **9**

D–H···A	Symmetry code	d(D–H) Å	d(H–A) Å	d(D–H) Å	<(D–H–A) °
**4**					
O3–H3···O7	*x*, -1+*y*, *z*	0.84	1.88	2.683(3)	159
O4–H4···O4	2-*x*, -1/2+*y*, 1-*z*	0.84	1.96	2.799(2)	175
O17–H17···O3	1-*x*, 1/2+*y*, 1-*z*	0.84	1.85	2.682(3)	176
**9**					
O1–H11···O3	*x*, 1+*y*, *z*	0.83	1.91	2.720(3)	168
O2–H21···O6	*x*, 1+*y*, *z*	0.81	1.98	2.775(3)	166
O3–H31···O1	1-*x*,-1/2 +*y*, -*z*	0.81	1.93	2.734(3)	173
O5–H51···O7	*x*, 1+*y*, *z*	0.83	1.96	2.752(3)	157
O6–H61···O2		0.82	1.98	2.782(3)	167
O7–H71···O5	1-*x*,-1/2 +*y*, 1-*z*	0.81	1.98	2.776(3)	171

ORTEP-like drawing of **4**

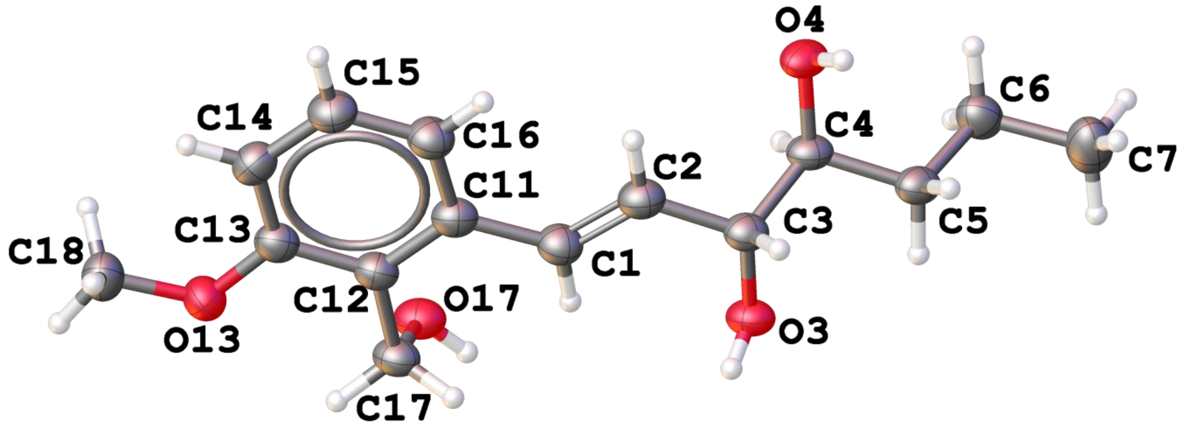

ORTEP-like drawing of **9**

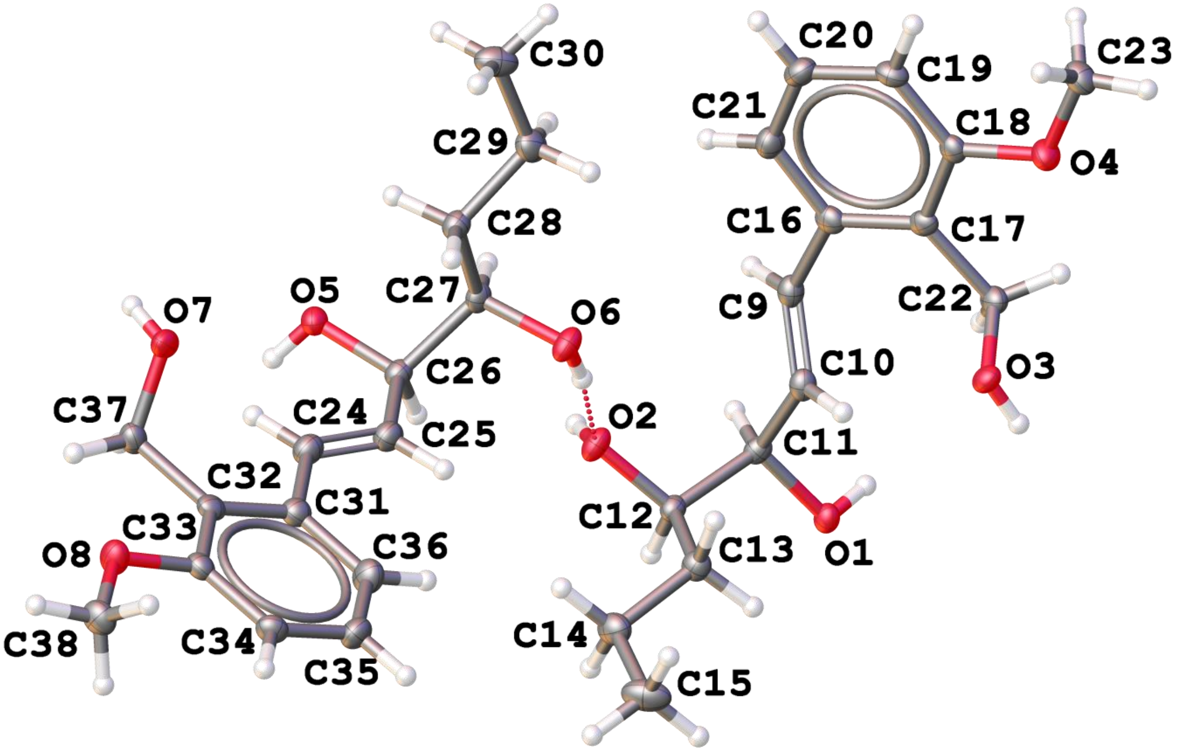

Hydrogen bond network in crystal structure of **4**

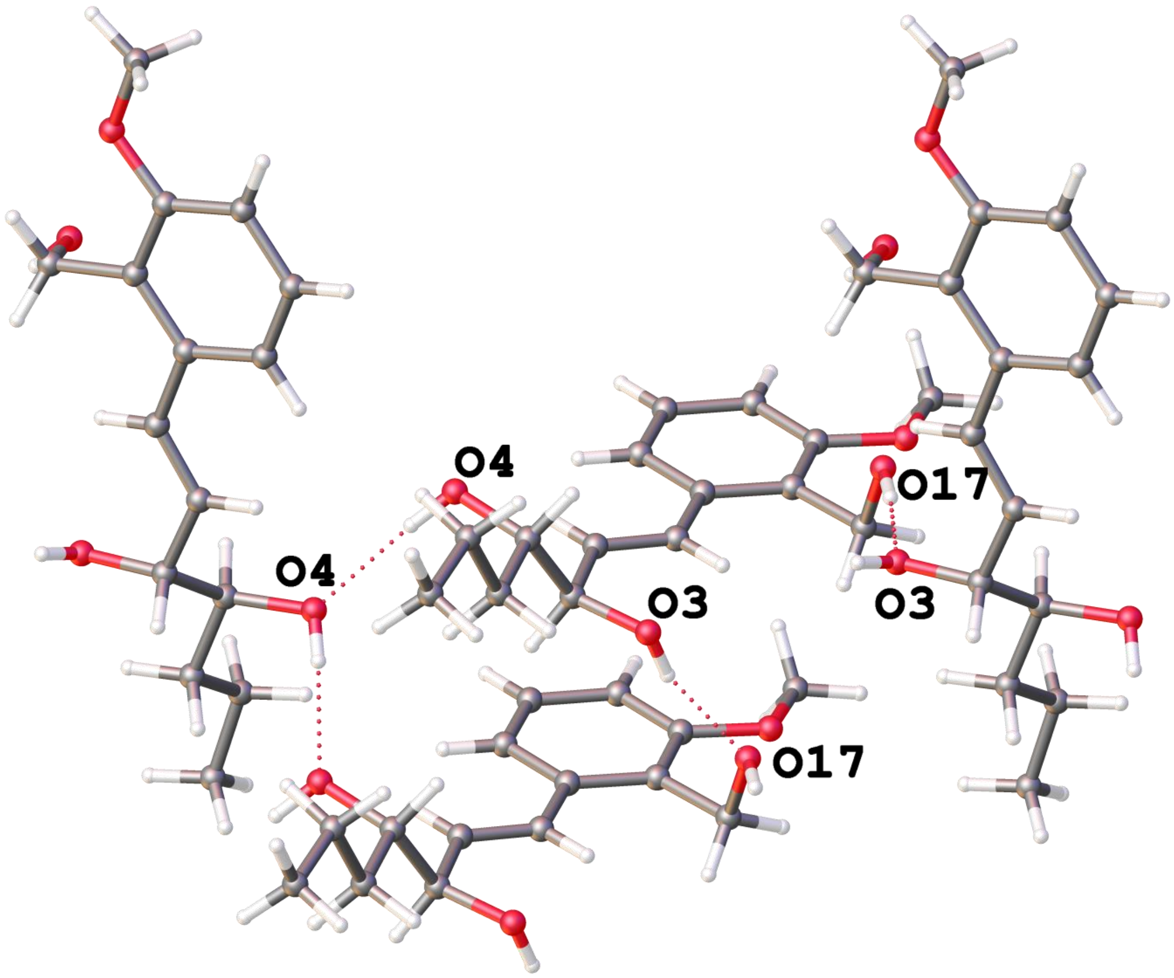

Hydrogen bond network in crystal structure of **9**

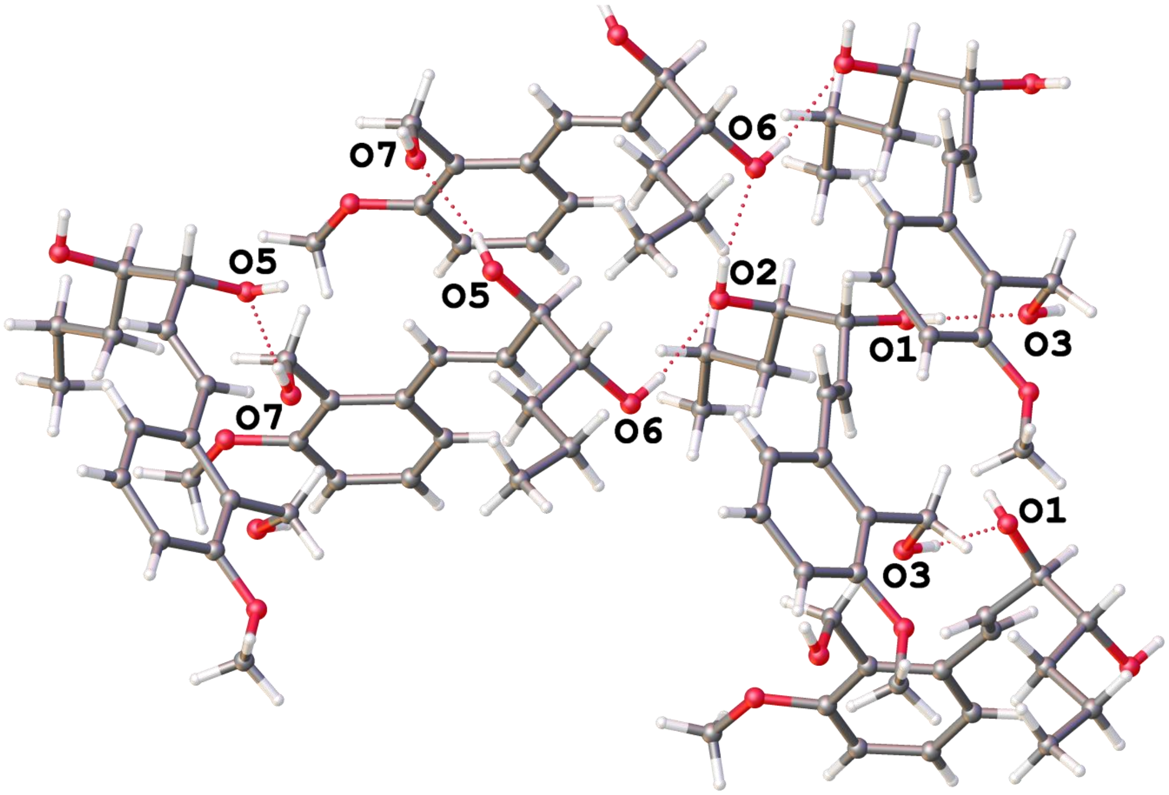

Supplement: Supplementary file 1 [file molecules-24-00862-s001.pdf]
